# Cost-effectiveness evidence on approved cancer drugs in Ireland: the limits of data availability and implications for public accountability

**DOI:** 10.1007/s10198-021-01365-2

**Published:** 2021-08-30

**Authors:** Suaad Almajed, Nora Alotaibi, Sana Zulfiqar, Zahraa Dhuhaibawi, Niall O’Rourke, Richard Gaule, Caoimhe Byrne, Aaron M. Barry, Dylan Keeley, James F. O’Mahony

**Affiliations:** School of Medicine Trinity College, 2-4 Foster Place, Dublin 2, Ireland

**Keywords:** Cost-effectiveness, Policy oversight, Resource allocation, Transparency, I18

## Abstract

**Background:**

We surveyed evidence published by Ireland’s National Centre for Pharmacoeconomics (NCPE) on the cost-effectiveness of cancer drugs approved for funding within the Irish public healthcare system. The purpose is threefold: to assess the completeness and clarity of publicly available cost-effectiveness data of such therapies; to provide summary estimates of that data; to consider the implications of constraints on data availability for accountability regarding healthcare resource allocation.

**Methods:**

The National Cancer Control Programme lists 91 drug-indication pairs approved between June 2012 and July 2020. Records were retrieved from the NCPE website for each drug-indication pair, including, where available, health technology assessment (HTA) summary reports. We assessed what cost-effectiveness data regarding approved interventions is available, aggregated it and considered the consequences of reporting constraints.

**Results:**

Among the 91 drug-indication pairs 61 were reimbursed following full HTA, 22 after a rapid review process and 8 have no corresponding NCPE record. Of the 61 where an HTA report was available, 41 presented costs and quality-adjusted life-year (QALY) estimates of the interventions compared. Cost estimates and corresponding incremental cost-effectiveness ratios (ICERs) are based on prices on application for reimbursement. Reimbursed prices are not published. Aggregating over the drug-indication pairs for which data is available, we find a mean incremental health gain of 0.85 QALY and an aggregate ICER of €100,295/QALY, which exceeds Ireland’s cost-effectiveness threshold of €45,000/QALY.

**Conclusion:**

Reimbursement applications by pharmaceutical manufacturers for cancer drugs typically exceed Ireland’s cost-effectiveness threshold, often by a considerable margin. On aggregate, the additional total net cost of new drugs relative to current treatments needs to be more than halved for the prices sought on application to be justified for reimbursement. Commercial confidentiality regarding prices and cost-effectiveness upon reimbursement compromises accountability regarding the fair and efficient allocation of scarce healthcare resources.

## Introduction

This study considers Ireland’s health technology assessment (HTA) framework for the assessment of cancer drugs and surveys the completeness of the cost-effectiveness evidence made public. It uses this assessment of the constraints on publicly available data to motivate a discussion of the implications for accountability regarding the allocation of scarce healthcare resources.

Cancer drugs are a particular class of intervention that present persistent challenges to achieving value for money for many healthcare systems [1]. Many countries conduct HTAs to determine if drugs provide sufficient value for money to merit reimbursement. HTAs combine systematic reviews, trial data and modelling to estimate the clinical and cost-effectiveness of candidate interventions. Policy makers consider these estimates alongside other considerations when forming their recommendations, such as budget impact and ethical concerns regarding access to care.

Ireland’s HTA framework for pharmaceuticals has been described and examined previously [2–8]. Ireland’s tax-funded public health system is managed by the Health Service Executive (HSE). The provision of pharmaceuticals by the HSE is subject to the 2013 Health Act, Schedule 3, Part 3 of which obliges the HSE to consider the cost-effectiveness and budget impact of candidate interventions alongside seven other points of consideration [9]. This is manifest in the requirement of manufacturers to submit a pharmacoeconomic evaluation (PE) to the HSE’s Corporate Pharmaceutical Unit when seeking approval for the reimbursement of new therapies.

The health economic decision criteria regarding new drugs in Ireland are set as part of an ongoing series of agreements between the pharmaceutical industry, the HSE and government, the most recent of which dates from 2016 [10]. An appendix to the agreement details what level of decision maker within the HSE can authorise approval of a new drug. This differs by levels of budget impact and cost-effectiveness, with the latter expressed at two thresholds of €20,000/quality-adjusted life-year (QALY) and €45,000/QALY. While the agreement does not articulate how these decision thresholds relate to the task of balancing the cost of new interventions with their opportunity cost, the €20,000/QALY and €45,000/QALY limits are widely interpreted as Ireland’s prevailing cost-effectiveness thresholds [7, 11–13]. Clearly only the upper threshold will ultimately be relevant if decisions can be escalated to a higher level within the HSE. The €45,000/QALY threshold is not binding in that drugs exceeding it are not necessarily rejected, but can be put forward for further consideration, which may include additional confidential price negotiations.

The National Centre for Pharmacoeconomics (NCPE) is the independent expert review body commissioned by the HSE to evaluate HTA submissions [14]. The NCPE conducts two tiers of analyses. A rapid review (RR) is a preliminary analysis of an information summary on the candidate technology presented by manufacturers containing clinical evidence and economic considerations including the treatment cost and anticipated budget impact [15–17]. There are several possible recommendations from the NCPE following a RR: that the intervention be forwarded for further consideration without conducting an HTA; a full HTA should be conducted due to questions regarding costs or cost-effectiveness; an HTA should not be conducted and the intervention should not be considered for reimbursement at the submitted price; finally, that a full HTA is not recommended until further data on either or both efficacy and safety is provided [18]. The criteria for a full HTA are described on the NCPE’s website [14]. They broadly correspond to an anticipated large budget impact, questionable clinical efficacy or potentially poor value for money.

On completing a full HTA the NCPE issues the Final Appraisal Report documenting their findings and a reimbursement recommendation to the HSE Drugs Group and publishes a summary of that report (henceforth referred to as an HTA summary) on the NCPE website [4]. The Final Appraisal Report is not made public. The NCPE can make four different post-HTA reimbursement recommendations: that the drug be considered for reimbursement at the assessed price; a conditional recommendation that the drug be considered for reimbursement if the price can be reduced in subsequent negotiations; a conditional recommendation that the drug not be considered for reimbursement unless the price can be reduced in subsequent negotiations; finally, simply to recommend against consideration for reimbursement [18]. Importantly, the second and third conditional recommendations explicitly refer to the possibility for post-submission price negotiations to achieve a better price. In all cases the NCPE also state that their recommendations should be considered within the criteria for reimbursement stated in the 2013 Health Act.

The National Cancer Control Programme (NCCP) is a directorate within the HSE responsible for population cancer control in Ireland [19]. Part of this function is to manage and deliver cancer care in collaboration with care providers. In the case of cancer drugs, the NCPE appraisal report will also be submitted to the NCCP’s Technology Review Committee. This committee considers the NCPE’s PE assessment and can issue one of three recommendations: rejection; adoption; or adoption subject to a price reduction [20]. This recommendation is issued to the NCCP’s Director, who then brings it to the HSE Drugs Group for consideration. If a cancer drug is approved by the HSE Drugs Group, it is then added to the list of approved cancer drugs maintained by the NCCP [21]. Meeting minutes from both the Technology Review Committee and the Drugs Group are published [22, 23].

The NCPE HTA summaries publish costs and ICERs on the basis of the list prices on application. If there are post-HTA price negotiations prior to approval, then these costs, ICERs and list prices will not be representative of the agreed prices on adoption. Although the meeting minutes of both the Technology Review Committee and the Drugs Group are published, the pricing details of approved drugs are redacted. This means there is no publicly available source for the agreed prices, associated costs and ICERs of cancer drugs on reimbursement.

Approved cancer drugs are funded under three schemes, the first of which is the community drug scheme of the Primary Care Reimbursement Services (PCRS), which funds self-administered drugs for community-resident patients. The second is the Oncology Drug Management System (ODMS) for high-cost drugs administered in hospitals. The final source is individual hospital budgets that sponsor drugs administered in hospitals.

Previous studies have assessed aspects of Ireland’s HTA appraisal process, including the choice of appraisal pathways, appraisal times and analyses of particular classes of therapies [3, 5, 7, 24]. The objective of this study is to survey the available evidence on the cost-effectiveness of publicly funded cancer drugs in Ireland and to assess the clarity, consistency and completeness of that data. A secondary objective is to use the available data to derive aggregate cost-effectiveness estimates for approved cancer therapies. The third objective is to use this appraisal of available cost-effectiveness data to inform a discussion on the implications of commercial confidentiality regarding reimbursed prices for accountability regarding the allocation of scarce healthcare funds. No previous study has provided such an analysis to our knowledge.

## Methods

We compiled a data set by combining publicly available sources on approved cancer drugs in Ireland. The primary source was the list of all approved treatments maintained by the NCCP [21]. As each drug can have multiple clinical indications we describe each separate drug and indication combination as a drug-indication pair. The NCCP’s list of approved drugs names and dates all drug-indication pairs approved since May 2012, details under which funding scheme reimbursement was made and provides links to NCCP regimen summary documents. The list was assessed in February 2020 and the search updated in July 2020.

We then consulted the NCPE website to find information for the same drug-indication pairs that the NCCP list as approved [25]. The website details what applications have been made for which drugs and for what indications, the dates of each initial application and reports if the drug-indication pair has been subject to RR alone or has undergone full HTA. The website also provides HTA summaries for those pairs subject to full assessment. Recently the NCPE has offered both plain English and technical HTA summary documents. Where both are available we refer to the technical summary.

Nine reviewers recovered data for the drug-indication pairs (RG, CB, AB, DK, NOR, SA, SZ, NA, ZD). Each reviewer was responsible for assessing a portion of the NCCP’s list and cross checking the data extraction of another reviewer.

Each drug-indication pair was categorised according to mechanism of action (MOA) and cancer types according to the International Classification of Diseases (ICD). Cancers were classified using the ICD 10 codes reported in the associated NCCP regimen summaries. Similarly, the drugs’ MOA were classified using the World Health Organisation (WHO) Anatomical Therapeutic Chemical (ATC) codes from the regimen summaries and cross-referenced to the ATC listings maintained by the WHO [26]. Some of the cancer and drug type categories were merged where numbers were small. The NCCP regimen summaries link to European Medicines Agency (EMA) product characteristics descriptions for each therapy, which were used to identify the market authorisation holder for each drug. The orphan status for each drug-indication pair was determined using the European Commission’s register of medicinal products and cross-referenced against the Orphanet database [27, 28].

For those drug-indication pairs in which a full HTA was conducted, the reviewers examined the NCPE HTA summaries. In some cases a single drug approval record on the NCCP website corresponds to multiple indications. We considered each pair separately unless the relevant NCPE HTA summary aggregated the cost-effectiveness of the indications together. In these cases we considered the multiple indications to correspond with a single drug-indication pair. Similarly, in cases in which additional subgroup analyses were presented alongside the primary patient group, we only consider the primary patient group. For those cases that are disaggregated between indications the NCPE HTA summaries typically report incremental cost-effectiveness ratios (ICERs) for the different indications separately but the report budget impact for the indications combined. Accordingly, our results are presented in the same way.

We extracted and reported information from the HTA summaries regarding the comparisons made between treatments, ICERs, budget impact and the NCPE recommendation. Note that the ICERs and budget impact from the HTA summaries relate to list prices, not final reimbursed prices. We retrieved the identity of the applicant firm from the summaries, which is not always synonymous with the market authorisation holder reported by the EMA. We recorded the date the HTA summary was published, the summary length and if the NCPE website reported post-assessment price negotiations were conducted for the drug-indication pairs. We also appraised the time taken to reimbursement as the difference in time in months between the first mention of the drug-indication pair on the NCPE website and the date of listing by the NCCP as an approved drug. Note this total reimbursement time not only includes the time taken for the NCPE to appraise the intervention, but also includes any additional time taken to receive clarifications or amendments from manufacturers to submissions and for any price negotiations subsequent to the NCPE’s appraisal.

Where health effects and ICERs were reported for both QALYs and life years gained, we recorded the outcomes for QALYs. Where summaries reported both the manufacturer-estimated outcomes and outcomes based on what the NCPE stated was the most plausible set of assumptions, we recorded the latter. Where the NCPE summaries reported ICERs based on both deterministic and probabilistic analysis, we recorded the latter. In some summaries, the base-case ICERs were explicitly reported, while in others, several ICERs were given for the intervention relative to various comparators. To identify a single ratio, we recorded the ICER based on a comparison to the current standard of care. Some summaries did not report what the current standard of care is. In these cases, we recorded the highest of the reported ratios as this corresponds with the ICER on the efficient frontier.

We recorded on what basis the budget impact was recorded within the HTA summaries. This included gross and net budget impact over either 1 or 5 years. In cases in which a budget impact is reported as a range, we recorded the midpoint of that range.

We assessed the costs of the interventions reported in the HTA summaries. Standard CEA practice is to base reimbursement decisions on the incremental differences between discounted treatment costs of alternative interventions. Under standard methods such costs are the total treatment costs net of any resulting changes in related care costs such as hospitalisation or treatment reoccurrence. Irish HTA guidelines recommend that analyses are conducted with a lifetime time horizon and are assessed using the health payer perspective [13]. While the NCPE HTA summaries did not typically explicitly state what the cost estimates relate to, we assumed the reported costs accord with standard CEA practice in Ireland.

We recorded the incremental costs and QALYs of the intervention of interest versus the relevant base case comparator where reported. In some instances, costs and QALYs were reported for some comparisons but not others. If the costs and QALYs for what appears the primary comparison of interest could be inferred from the reported figures from other comparisons, we used these. In other cases, the incremental costs and QALYs corresponding with the NCPE’s preferred parameter set were not reported. In these cases, we recorded the incremental costs and QALYs reported for the most relevant scenario for which outcomes were reported. In each drug-indication pair, the recorded ICER, costs and QALYs all correspond to the same incremental comparison.

We conducted a descriptive analysis of the compiled data to demonstrate the completeness of the publicly available evidence and to provide an overview of the relationships between the variables recorded. We compiled the unweighted arithmetic mean of the costs, QALYs, budget impact and ICERs. We also computed a weighted arithmetic mean of the incremental costs and QALYs weighted by the reported 5-year gross budget impact and calculated a weighted aggregate ICER from this.

We use the analysis of the published data and aggregate cost-effectiveness estimates presented in the following results section to inform a discussion regarding the implications of data availability for considerations of accountability regarding healthcare resource allocation.

## Results

Appendix Tables 4, 5, 6, 7, 8 provide detailed records of each drug-indication pair assessed. Table 4 records the drug name, approved indication, approval date and the internet addresses of both the NCCP regimen listing and record on the NCPE website. Table 5 details the drugs’ proprietary names, market authorisation holders, ICD10 codes and mechanism of action. It also records if the indication includes metastatic disease and the drugs’ current orphan status. In two instances it is known that the applicant firm is not synonymous with the market authorisation holder. The applicants for dabrafenib and ponatinib are GlaxoSmithKline and ARIAD Pharmaceuticals, respectively. Table 6 details aspects of the appraisal process, including at what stage in the NCPE’s appraisal was the drug recommended for consideration for reimbursement and under what funding pathway it was reimbursed, the total time taken to from application to reimbursement, the length of the HTA summary (if applicable) and whether the NCPE website reports if reimbursement was made following post-assessment price negotiations or not. Table 7 details the information extracted from HTA summaries. This includes the basecase ICER, incremental costs and QALYs, 5-year gross budget impact and records if costs and QALYs were reported for all the treatment strategies mentioned within the summary. Table 8 reports the number of approved drug-indication pairs by market authorisation holder and the associated total 5-year gross budget impact where reported within the NCPE HTA summaries.

The NCCP list of approved cancer therapies contained 77 drugs, drug combinations or distinct drug dosages as approved between May 2012 and July 2020. Following the separation and merging of indications described in the methods, there are 91 drug-indication pairs in total. While most drugs only have one approved indication, the four most commonly approved drugs (including combination therapies) are nivolumab, idelalisib, ponatinib and pembrolizumab with 7, 5, 4 and 4 approved indications, respectively. The NCPE website reports reimbursement was made following post-appraisal price negotiations in 65 of the 91 drug-indication pairs.

NCPE HTA summaries are only published for drug-indication pairs subject to full HTA review. There were 61 pairs with published HTA summaries available. The summaries range from 3 to 8 pages long, have a mean length of approximately 6 pages and have increased in length modestly over the period assessed.

We found 8 pairs for which there is no clear record of a cost-effectiveness appraisal on the NCPE website, either RR or full HTA. There was no mention of siltuximab (pair 28) on the NCPE website at all at the time of analysis. In the remaining 7 cases (pairs 18, 19, 22, 37, 44, 47 and 48), there were records of the drugs, but not for the indications in question.

While there were 61 pairs with HTA summaries, not all reported health economic outcomes. ICERs or an outcome of dominance were reported in all 61, but relevant incremental costs and QALYs were only reported in 41 summaries. There were 48 summaries that reported the 5-year gross budget impact, yielding a total of €1.2Bn. As gross budget impact does not account for possible substitutions of older therapies by new drugs in the same indication, the €1.2Bn total will exceed the net budget impact at the list prices on application. There were a further 8 studies that reported a 1-year budget impact.

We assessed the sample of pairs for which HTA summaries are available to assess how the completeness of data changed over time. In particular, we assessed what proportion of the sample included any two or more of an estimate of incremental costs, incremental QALYs or a 5-year budget impact (either gross or net). Dividing the sample in two chronologically according to the date of completion of the NCPE HTA review, 52% of the sample included any two or more data items for the first half of the sample, rising to 77% for the second half of the sample. While only a crude measure, this indicates that the completeness of reporting of cost-effectiveness data within the NCPE HTA summaries has increased over time.

**Fig. 1:**
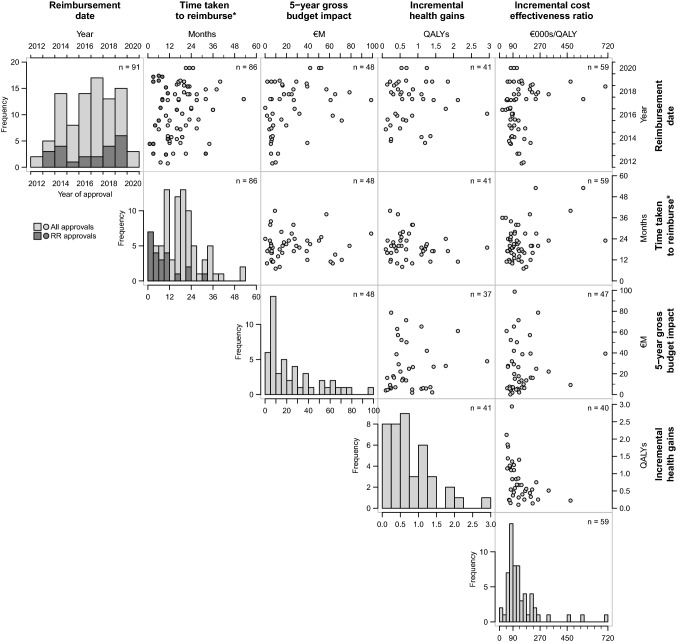
Correlation matrix of selected metrics of appraisal. *Total time between initial listing on the NCPE website and listing by the NCCP as approved

Figure 1 shows a correlation matrix of a selection of data from this analysis compiled with a series of histograms on the diagonal. The bars in the histogram and the points in the scatter plots shaded light grey correspond to approvals following a full HTA, those in dark grey correspond to approvals without full HTA. The sample size in each plot varies as the number of available data points varies (given as n in each plot). The first histogram shows the distribution of approvals over time. The first approval listed by the NCCP is in May 2012 and the most recent in July 2020. While there is no distinct trend in approvals, clearly more have been approved annually from between 2016 and 2019 than earlier in the sample. Although the number of approvals without requiring full HTA has been few, the number increased in 2018 and 2019.

Table 1 summarises at what stage of the HTA appraisal process had a drug reached prior to approval and under what funding pathway was the drug reimbursed. The table also details the time taken to reimburse in months. Of the 91 pairs, 3 were recommended for consideration at RR and subsequently approved. A further 19 were subsequently approved following a RR, but without progressing to full HTA. In these cases, a recommendation for consideration for reimbursement was not made at RR, but it appears subsequent negotiation avoided the need for a full HTA, even if recommended by the NCPE. Similarly, there were 3 and 58 recommendations at or after the full HTA stage, respectively. The three pairs recommended for consideration at HTA were aflibercept, dabrafenib and pembrolizumab as first line monotherapy for melanoma (pairs 14, 21, 34, respectively). This indicates that only a small minority of applications are considered sufficiently cost-effective to recommend consideration for reimbursement at the full HTA stage and most required further negotiation before they could progress to subsequent approval.

**Table 1:** Reimbursement time according to appraisal pathways and funding stream

	Drug-indication pairs, *n*	Mean time taken to reimburse^a^, months
Appraisal pathway		
At RR	3	6
After RR	19	10
At HTA	3	11
After HTA	58	22
Undocumented	8	NA
Total	91	18
Funding stream		
PCRS	53	19
ODMS	37	18
Individual hospital budgets	1	12
Total	91	18

^a^Total time between initial listing on the NCPE website and listing by the NCCP as approved

The overall mean time taken from application to reimbursement is 18 months. The mean reimbursement time appears shorter under RR than full HTA. Similarly, the reimbursement time for those pairs approved following post-HTA negotiations is longer than those at the other appraisal stages. The second histogram in Fig. 1 shows that most approvals are made in less than 24 months, but there are some outliers at over 4 years. The distribution of RR approvals is shown in dark grey, most of which are within 12 months. As mentioned in the methods, the total time to reimbursement assessed here is not synonymous with the time taken to appraise a given intervention, a detailed description and examination of which in an Irish context is given by Connolly et al. [3].

Somewhat over half of the approved drug-indication pairs are reimbursed under the PCRS. Only one pair (14—aflibercept) was listed for funding by individual hospital budgets. The remaining approvals were under the ODMS. There is no apparent difference in the reimbursement time between PCRS and ODMS funded drugs.

Table 2 summarises the approved pairs according to their therapeutic class and mechanism of action and contains aggregate estimates of budget impact, incremental costs and QALYs and ICERs from the HTA summaries. Lymphomas, leukaemias and lung cancer are the top three disease categories by number of approvals. They count for half of all approvals between them. Of the total approved indications, 48 included metastatic disease. Regarding the therapeutic categories, protein kinase inhibitors and monoclonal antibodies are the two notably large groups, accounting for two thirds of all approvals. At the time of investigation, 11 pairs held positive orphan status.

**Table 2:** Health economic outcomes by disease and treatment characteristics

	Drug-indication pairs, n	Total 5-year gross budget impact, €M	Mean 5-year gross budget impact, €M	Mean incremental costs, €	Mean incremental effects, QALYs	Mean ICERs, €/QALY
Disease category						
Breast	7	212	35	65,695	0.37	164,634
Digestive organs	6	14	5	12,872	0.16	88,082
Kidney	4	43	22	66,035	0.89	132,010
Lung	13	245	35	81,862	0.59	141,323
Prostate	6	93	23	85,050	0.58	126,313
Skin	15	300	30	99,296	1.15	171,566
Leukaemias	18	124	21	111,723	1.27	99,794
Lymphomas	9	48	12	55,687	0.77	78,973
Other blood cancers	6	99	25	68,887	0.58	264,864
Other	7	5	5	88,870	1.02	90,750
Total	91	1182	25	79,231	0.80	141,393
Metastatic disease	48	820	29	76,836	0.74	128,288
Therapeutic category						
Antimetabolites, plant alkaloids, cytotoxic antibiotic and related substances	6	10	5	10,553	0.15	96,976
Immunomodulating agents and other non-antineoplastic therapies	9	108	22	61,259	0.50	114,915
Monoclonal antibodies	28	614	31	65,993	0.90	118,796
Protein kinase inhibitors	34	304	22	96,492	0.71	141,084
Other antineoplastic agents	14	145	24	105,899	1.18	226,618
Total	91	1182	25	79,231	0.80	141,598
Orphan status	11	114	16	86,248	0.88	141,773

The number of approvals in the reported disease and therapeutic categories is small in many cases. Accordingly, it is important not to over-interpret the estimates disaggregated by category. The three largest disease categories in terms of gross budget impact were skin, lung and breast cancer, which together account for almost two thirds of the total. Naturally, the total budget impact depends in part on the number of approvals, so we also present the mean budget impact per approval within each category. While the disparities in mean budget impact are smaller, the same three categories still carry the three largest budget impacts. These mean figures still reflect, in part, the anticipated patient population size within each category. The mean incremental cost provides a per-patient estimate of the discounted net incremental cost of care and, therefore, does not reflect the size of the indications. On this basis, skin, lung and breast cancer do not appear notably different from the others. Similar conclusions apply to the therapeutic categories, in that the very large total budget impact of monoclonal antibodies and protein kinase inhibitors appear largely to reflect the number of approvals as the mean budget impact and incremental costs much more aligned with the overall means. While the mean budget impact of metastatic disease is effectively the same as the overall mean, orphan treatments appear to have a lower mean budget impact.

The third histogram shown in Fig. 1 shows the heavily right skewed distribution of 5-year gross budget impact, with clear mode between €5–10 M. Almost 60% of approvals have an estimated 5-year gross budget impact less than €20 M, while approximately 20% have a budget impact greater than €40 M.

Table 3 reports selected findings from the analysis including aggregated outcomes. The range of incremental health gains reported is 0.10 to 2.94 QALYs, with an unweighted mean of 0.80. An unweighted mean of the reported ICERs is €141,598/QALY. The estimated weighted mean intervention cost, QALY gains and resulting aggregate ICER when weighting according to the gross 5-year budget impact are €85,164, 0.85 and €100,295/QALY, respectively. Note that the unweighted and weighted ICERs differ in part because not all HTA summaries report budget impact or costs and QALYs, so the weighted values are based on fewer studies (the unweighted mean ICER from pairs used to determine the weighted aggregate ICER is 133,843/QALY, which is less than the overall unweighted mean). The distribution of incremental health effects in Fig. 1 shows most are below 0.75 QALYs, but only very few exceed 1.5 QALYs. The distribution of ICERs shown in Fig. 1 shows few below the €45,000/QALY threshold, a cluster between 2 and 3 times the threshold and several very high outliers. Overall, of the 61 drug-indication pairs for which full HTA summaries are available 5 (8%) either have ICERs within Ireland’s threshold or are more effective and less costly than their comparators, while the remaining 56 (92%) have ICERs above the threshold.

Overall, the correlation plots indicate few notable relationships. There is a negative association between the QALYs gained and ICERs, but it is not pronounced. There may be some indication that the variance in appraisal duration, budget impact and ICERs are increasing over time (Table 3).

**Table 3:** Aggregate cost and effects estimates

	5-year gross budget impact, €M	Incremental costs, €	Incremental health effects, QALYs	Basecase ICER, €/QALY
Minimum	0.22	− 3092	0.10	20,000
Maximum	98.80	243,725	2.94	703,426
Unweighted mean	24.62	79,231	0.80	141,598
Weighted mean	–	85,164	0.85	100,295

## Discussion

We need to explicitly acknowledge this study's main limitation at the outset of the discussion, namely, that we only have access to the drug costs, budget impact and ICERs corresponding to the list prices on application for reimbursement, not the actual agreed prices paid following negotiation. The agreed prices will be lower than those on application in most cases, although by what margin we cannot know. Furthermore, the effective prices will also be lower due to rebates and risk sharing schemes.

The following discussion describes what is useful about the currently reported cost-effectiveness estimates. We then outline some of the inconsistencies and other reporting gaps we observed in the NCPE summaries. Finally, we consider the implications of constraints on complete reporting for accountability regarding resource allocation.

### Usefulness of NCPE summaries

Although the list prices and ICERs published in the HTA summaries are not informative of actual resource allocation decisions, NCPE summary publications do still provide useful information. First, publishing evidence of ICERs breaching the threshold reveals the list prices sought by manufacturers will lead to aggregate net harm to the health system. We found that 92% of the drug indication pairs for which information was available were not cost-effective at the prices on application. This provides justification for the sometimes long negotiating periods required to agree prices. Moreover, the published ICERs give an indication of how much costs need to be moderated relative to those at list prices. The weighted aggregate ICER from our analysis of just over €100,000/QALY is more than twice the current threshold. We see that the additional total net costs of new cancer drugs at list prices need to be reduced, on aggregate, by more than half if reimbursement is not to represent an unfair and ethically questionable use of resources (assuming that the QALYs gained treating cancer are of equal value as those foregone by other patients). Indeed, that interpretation rests on the assumption the current €45,000/QALY threshold is a fair representation of the opportunity cost of other care in Ireland. Since it is arguably a substantial underestimate [29, 30], the additional total net costs would need to be very substantially less than half than those at list prices for new cancer drugs to avoid being net damaging to the Irish health system.

Another important benefit offered by the HTA summaries is that they provide evidence on other aspects of the HTA appraisal, such as the treatment comparisons considered, the size of anticipated health gains and some indication of the strength of evidence presented. Public discourse on novel drugs often features descriptions of "game changers", "breakthrough" treatments or other such terms [16, 17]. While of course some drugs may achieve complete remission in some patients, we believe it important the public appreciate that new cancer drugs approved in Ireland over nearly a decade will provide a mean of 0.85 QALYs; less than 1 year equivalent of good health. A sober assessment of the health gains offered by new treatments may inform a more balanced consideration of the choices between new drugs and the great many other interventions that the Irish health service struggles to provide timely access to [30].

### Inconsistencies and reporting gaps

We noticed some inconsistent reporting within the NCPE HTA summaries. Budget impact was not reported on a consistent basis, as it was variously reported in gross or net terms and the timeframe varied between summaries. Similarly, the anticipated patient population size was not always reported. The incremental costs and QALYs were not reported in all summaries and were also not always reported for all strategies mentioned in the summaries, thus precluding comparisons between strategies other than those reported by the NCPE. Overall, however, while not formally assessed against any objective criteria, we consider the consistency of reporting to be high across the NCPE HTA summaries. Moreover, as noted in the results, we find the completeness of reporting of costs, QALYs and budget impact within the HTA summaries to have increased over time.

One specific issue we noticed within the HTA summaries was the reporting of multiple ICERs for a given strategy based on comparisons to multiple interventions. A narrow textbook interpretation of CEA would suggest that this is incorrect and that any given intervention should only have one ICER on the efficient frontier. Moreover, the reporting of multiple ICERs including those based on comparisons to dominated strategies could cause confusion to decision makers regarding an intervention's cost-effectiveness. In practice, there may often not be a single relevant comparator. The current standard of care may vary between patients and depend on clinical judgement. Accordingly, the reporting of multiple ICERs is not necessarily reason for concern.

While the choice of comparator may be context-dependent it still deserves scrutiny. We found only five drug-indication pairs that were either dominant or had ICERs within the threshold (pairs 34 and 40–43). In the example of pair 34 pembrolizumab was dominant relative to the comparator of ipilimumab which itself was previously found to be cost-ineffective. Accordingly, pembrolizumab would not have been found to be cost-effective had the previous approval not exceeded the cost-effectiveness threshold. Accepting comparisons against cost-ineffective comparators prompts concerns of a spiral of increasing cost-ineffectiveness. If decision makers permit cost-ineffective choices to accumulate on top of each other, this will result in ever less efficient treatment within each indication. Accordingly, there may need to be more critical examination of the treatment status quo.

The RR process is a pragmatic way to triage the analytical workload of assessing new interventions and avoiding a full HTA process. A drawback of the current RR process is that no summary information is published on interventions appraised under this pathway. It seems reasonable that at least some of the information considered at RR could be published.

A small proportion of drug-indication pairs have no corresponding NCPE record. While they are few and the indications may be narrow, it is nevertheless disconcerting that at least some approvals appear to have effectively bypassed the controls applied to other interventions.

Our conclusion regarding the availability of data is that the NCPE HTA summaries provide a largely consistent, if not fully complete record of the costs and QALYs of the interventions on application. While there are evidence gaps arising from the lack of any reporting for interventions approved following RR and some concerns about ICER estimates based on comparisons to cost-ineffective technologies, the largest concern remains the unavailability of agreed prices and ICERs on approval.

### Implications for accountability

Withholding agreed prices because of commercial confidentiality is an understandable consequence of price negotiation between the State and manufacturers. This confidentiality is valuable to manufacturers for their negotiations in other markets [31]. Offering confidentiality to manufacturers may help decision makers secure savings for the State and is common practice in many countries [32, 33]. It is also suggested that price confidentiality might offer broader benefits in facilitating what is known as price discrimination, thereby permitting access to more countries on a pricing basis that reflects variation in ability to pay than would be achieved if common prices were paid by all nations [34]. A cost of such price confidentiality is compromised accountability regarding public health expenditure. Without actual agreed prices, there is no meaningful assessment that external parties can make of healthcare resources allocation, effectively protecting decision makers from scrutiny. This is concerning, as many interests including clinicians, patient advocacy groups, politicians and manufacturers will seek to call attention to the benefit of new treatments, while insufficient attention may be given to the countervailing costs to other patients.

The lack of meaningful accountability is particularly concerning given the mixed performance of Ireland’s healthcare system when compared internationally. Aspects of the Irish health system perform well, evidenced by rapid improvements in life expectancy and better than average performance in amenable mortality estimates relative to other EU nations [35]. Ireland also performs well on composite measures of health outcomes in general and on cancer mortality in particular [36]. Despite this, the Irish health system performs poorly in terms of access as it both lacks universal coverage and exhibits long waiting lists for elective care and has been ranked lowest out of 35 European nations with respect to waiting times [35, 36]. Indeed, the fact that the Irish health system provides demonstrably poor access to basic and cost-effective services is indicative that resources are not being used most efficiently [29].

A potential solution to this accountability problem would be the publication of some form of aggregated cost-effectiveness metrics based on reimbursed prices. The aggregated costs and QALYs presented in this analysis are an example of such reporting. The estimated incremental costs and QALYs aggregated over all interventions newly adopted each year could be published annually. Alternatively, it would be possible to publish the number of interventions reimbursed within intervals of multiples of the threshold as previously suggested by O’Mahony and Coughlan [29] and exemplified by the final histogram in Fig. 1 of this analysis. Either proposal would offer a degree of accountability regarding the allocation of health spending without revealing the agreed price paid for any given drug.

The above proposals would provide evidence of the effectiveness of current appraisal and price negotiation systems. It may be the case that current processes are highly effective at achieving value for money. The reporting proposal would give due credit without publishing confidential prices. Some may contend the onus should be on pharmaceutical manufacturers to be more transparent regarding the prices they charge. Indeed, the World Health Organisation has issued a resolution urging greater price transparency [37]. Despite this, we perceive the responsibility for compiling and publishing such analyses most naturally falls on public regulators rather than private commercial entities: in this case, that part of the decision-making process that is privy to the prices on reimbursement, namely, the HSE Drugs Group. While this manuscript has considered confidentiality regarding prices of cancer therapies, the same concerns and potential solutions apply to all drug spending.

We have already noted the primary limitation of this study, but there are others to acknowledge. We only assessed approved cancer drugs. Necessarily the analysis excludes drug-indication pairs that were assessed by the NCPE but ultimately rejected. Similarly, our analysis can only assess officially approved drugs and cannot appraise off-label use. Furthermore, the health economic outcomes extracted from the 61 pairs subject to HTA are not likely to be representative of the other interventions approved after RR, which will likely have both lower budget impact and ICERs on aggregate. In cases in which two budget impact estimates were published, we formed a point estimate by taking the mid-point between the published values. Such an estimate does not account for any possible skewness in costs. We have not conducted a statistical analysis of the outcomes recorded within our review, partly because the sample sizes within the disease and therapeutic categories are too small in most cases, but also because we consider the value of such an analysis questionable without access to the actual agreed prices. Our analysis reports the number of drug-indication combinations for which the NCPE record reimbursement was made following post-appraisal price negotiations. Given that the NCPE is not a party to such negotiations we do not know if the NCPE’s reporting of such negotiations is exhaustive or not.

Finally, a concluding note regarding data availability on agreed prices in Ireland. Like other research [24, 38], this analysis could only use list prices on application. Notably, a recent study investigating the potential of value of information analysis regarding cancer drugs in Ireland did have the benefit of access to confidential agreed prices [11]. Accordingly, there is precedent for the use of confidential pricing information in Ireland for research purposes.

## Conclusion

To date there has been no single analysis of the cost-effectiveness of cancer drugs in Ireland. Our analysis shows that each new approval for which data is published yields approximately 0.85 QALYs on average. Appreciating the size of this health gain may provide the public with a more realistic expectation of new cancer therapies. The aggregate ICER on application of approximately €100,000/QALY indicates that the additional costs of new drugs relative to existing therapies would need to be more than halved for reimbursement at list prices to be a fair and ethically justifiable use of scarce health resources. While the current publication of HTA summaries by the NCPE provide much useful data, additional reporting is required to accommodate commercial confidentiality while delivering meaningful accountability regarding decision maker choices regarding drug spending in Ireland. The most suitable body to provide such reporting is the HSE Drugs Group.

## Data Availability

All of the data used in this study are already in the public domain.

## Appendix

See Tables 4, 5, 6, 7, 8.

**Table 4:** Drug-indication pairs and data sources

Pair number	Drug name	Indication	NCCP URL^a^	NCPE URL^b^	Date reimbursed
1	Ipilimumab	Adults with advanced (unresectable or metastatic) malignant melanoma	Melanoma/105.pdf	Drugs/ipilimumab-yervoy/	May 2012
2	Abiraterone	Metastatic castration resistant prostate cancer which has progressed on or after a docetaxel-based chemotherapy regimen	Genitourinary/103-abiraterone-and-prednisolone-therapy.pdf	Drugs/abiratone-acetate-zytiga/	Jun 2012
3	Tegafur/gimeracil/oteracil	Advanced gastric cancer in combination with cisplatin	Gastrointestinal/235-cisplatin-and-teysuno%C2%AE-28-day-cycle.pdf	Drugs/tegafurgimeraciloteracil-teysuno/	Feb 2013
4	Axitinib	Adults with advanced RCC after failure, on a previous line of therapy, i.e., treatment with SUNItinib, or a cytokine	Genitourinary/axitinib104.pdf	Drugs/axitinib-inlyta/	Mar 2013
5	Cabazitaxel	Metastatic castration resistant prostate cancer previously treated with docetaxel containing regimen	Genitourinary/cabazitaxelprotocol.pdf	Drugs/carbazitaxel-jevtana-for-prostate-cancer/	Mar 2013
6	Mifamurtide	High-grade resectable non-metastatic osteosarcoma after macroscopically complete surgical resection, in children, adolescents and young adults	Sarcoma/mifamurtide.pdf	Drugs/mifamurtide-mepact/	Mar 2013
7	Vemurafenib	Adults with BRAF V600 mutation-positive unresectable or metastatic melanoma	Melanoma/102-vemurafenib-monotherapy-regimen.pdf	http://www.ncpe.ie/drugs/vermurafenib-zelboraf/	Mar 2013
8	Afatinib	As monotherapy for EGFR TKI-naïve adults with locally advanced or metastatic non-small cell lung cancer (NSCLC) with activating EGFR mutation(s)	Lung/221.pdf	Drugs/afatinib-giotrif/	Jan 2014
9	Bosutinib	Adults with chronic phase, accelerated phase, and blast phase Ph + CML previously treated with one or more TKI(s) and for whom imatinib, nilotinib and dasatinib are not considered appropriate treatment options	Leukemia-bmt/224-bosutinib-monotherapy-regimen.pdf	Drugs/bosutinib-bosulif/	Jan 2014
10	Decitabine	Adults aged 65 years and above with newly diagnosed de novo or secondary AML, according to the WHO classification, who are not candidates for standard induction chemotherapy	Leukemia-bmt/231.pdf	Drugs/decitabine-dacogen/	Jan 2014
11	Eribulin	LABC or MBC which has progressed after at least two chemotherapeutic regimens for advanced disease. Prior therapy should have included an anthracycline and a taxane unless patients were not suitable for these treatments	Breast/228-eribulin-monotherapy.pdf	Drugs/eribulin-halaven/	Jan 2014
12	Pertuzumab	Adults with HER2-positive MBC or locally recurrent unresectable breast cancer, who have not received previous anti-HER2 therapy or chemotherapy for their metastatic disease	Breast/350%20pertuzumab%20and%20trastuzumab%20and%20chemotherapy.pdf	Drugs/pertuzumab-perjeta/	Feb 2014
13	Ruxolitinib	Disease-related splenomegaly or symptoms in adults with post polycythaemia vera myelofibrosis	Leukemia-bmt/229-ruxolitinib-monotherapy-regimen.pdf	Drugs/ruxolitinib-jakavi/	Feb 2014
		Disease-related splenomegaly or symptoms in adults with primary myelofibrosis (chronic idiopathic myelofibrosis)			
		Disease-related splenomegaly or symptoms in adults with post essential thrombocythaemia myelofibrosis			
14	Aflibercept	Combination with irinotecan/5-fluorouracil/folinic acid (FOLFIRI) chemotherapy in adults with metastatic colorectal cancer that is resistant to or has progressed after an oxoliplatin-containing regimen	Gastrointestinal/238-aflibercept-and-folfiri-therapy-14-days.pdf	Drugs/aflibercept-zaltrap/	Apr 2014
15	Crizotinib	Adults with previously treated anaplastic lymphoma kinase (ALK)-positive advanced non-small cell lung cancer (NSCLC)	Lung/243-crizotinib-monotherapy-regimen.pdf	Drugs/crizotinib-xalkori/	Jun 2014
16	Vandetanib	Aggressive and symptomatic medullary thyroid cancer (MTC) in patients with unresectable locally advanced or metastatic disease	Headandneck/242vandetanibmonotherapy.pdf	Drugs/vandetanib-caprelsa/	Jun 2014
17	Brentuximab vedotin	Adults with relapsed or refractory CD30+ Hodgkin lymphoma (HL): following autologous stem cell transplant (ASCT)	Lymphoma-myeloma/234-brentuximab-vedotin-monotherapy-regimen.pdf	Drugs/brentuximab-vedotin-adcetris/	Aug 2014
18	Brentuximab vedotin	Adults with relapsed or refractory CD30+ Hodgkin lymphoma (HL): following at least two prior therapies when ASCT or multi-agent chemotherapy is not an option	Lymphoma-myeloma/234-brentuximab-vedotin-monotherapy-regimen.pdf	Drugs/brentuximab-vedotin-adcetris/	Aug 2014
19	Brentuximab vedotin	Adults with relapsed or refractory systemic anaplastic large cell lymphoma (sALCL)	Lymphoma-myeloma/234-brentuximab-vedotin-monotherapy-regimen.pdf	Drugs/brentuximab-vedotin-adcetris/	Aug 2014
20	Enzalutamide	Adults with metastatic castration-resistant prostate cancer whose disease has progressed on or after docetaxel	Genitourinary/233-enzalutamide-monotherapy.pdf	Drugs/enzalutamide-xtandi/	Aug 2014
21	Dabrafenib	Adults with unresectable or metastatic melanoma with the BRAF V600 mutation	Melanoma/237dabrafenib.pdf	Drugs/dabrafenib-tafinlar/	Sep 2014
22	Regorafenib	Adults unresectable or metastatic gastrointestinal stromal tumours (GIST) who progressed on or are intolerant to prior treatment with imatinib and sunitinib	Sarcoma/244-regorafenib-monotherapy.pdf	Drugs/regorafenib-stivarga-for-gist/	Apr 2015
23	Regorafenib	Adults with metastatic colorectal cancer (mCRC) who have been previously treated with, or are not considered candidates for, available therapies. These include fluoropyrimidine-based chemotherapy, anti-VEGF and anti-EGFR therapies	Sarcoma/244-regorafenib-monotherapy.pdf	Drugs/rigorafenib-stivarga/	Apr 2015
24	Abiraterone	Metastatic castration resistant prostate cancer in men who are asymptomatic or mildly symptomatic after failure of androgen deprivation therapy in whom chemotherapy is not yet clinically indicated	Genitourinary/103-abiraterone-and-prednisolone-therapy.pdf	Drugs/abiratone-acetate-zytiga-for-mcrpc-post-adt/	May 2015
25	Radium 223	Adults with castration-resistant prostate cancer, symptomatic bone metastases and no known visceral metastases	Genitourinary/257-radium-223-therapy1.pdf	Drugs/radium-223-xofigo/	May 2015
26	Obinutuzumab	Combination with chlorambucil for adults with previously untreated chronic lymphocytic leukaemia (CLL) and with comorbidities making them unsuitable for full-dose fludarabine based therapy	Leukemia-bmt/286 obinutuzumab-and-chlorambucil-therapy.pdf	Drugs/obinutuzumab-gazyvaro/	Aug 2015
27	Pixantrone	Monotherapy for adults with multiply relapsed or refractory aggressive Non-Hodgkin B-cell Lymphomas (NHL)	Lymphoma-myeloma/255-nccp-pixantrone-ver2final-.pdf	Drugs/pixantrone-pixuvri/	Aug 2015
28	Siltuximab	Adults with Multicentric Castleman’s disease (MCD) who are human immunodeficiency virus (HIV) negative and human herpes virus 8 (HHV-8) negative	Lymphoma-myeloma/277-nccp-siltuximab-ver2final-.pdf	NA	Aug 2015
29	Trastuzumab Emtansine	Adults with HER2-positive, unresectable locally advanced or metastatic breast cancer who previously received trastuzumab and a taxane, separately or in combination. Patients should have either: received prior therapy for locally advanced or metastatic disease, or developed disease recurrence during or within 6 months of completing adjuvant therapy	Breast/206.pdf	Drugs/trastuzumab-emtansine-kadcyla/	Aug 2015
30	Enzalutamide	Metastatic castrate resistant prostate cancer in men who are asymptomatic or mildly symptomatic after failure of androgen deprivation therapy (ADT) in whom chemotherapy is not yet clinically indicated	Genitourinary/233-enzalutamide-monotherapy.pdf	News/enzalutamide-xtandi-pre-chemotherapy/	Jan 2016
31	Lenvatinib	Adults with progressive, locally advanced or metastatic, differentiated (papillary/follicular/Hürthle cell) thyroid carcinoma (DTC), refractory to radioactive iodine	Headandneck/295.pdf	Drugs/lenvatinib-lenvima/	Jan 2016
32	Nab-Paclitaxel	Combination with gemcitabine for the first-line treatment of adults with metastatic adenocarcinoma of the pancreas	Gastrointestinal/256.pdf	Drugs/nab-paclitaxel-abraxane/	Feb 2016
33	Pomalidomide	Combination with dexamethasone for adults with relapsed and refractory multiple myeloma who have received at least two prior treatment regimens, including both lenalidomide and bortezomib, and have demonstrated disease progression on the last therapy	Lymphoma-myeloma/245-pomalidomide-and-dexamethasone.pdf	Drugs/pomalidomide-imnovid/	Feb 2016
34	Pembrolizumab (200/400 mg)	First line monotherapy for advanced (unresectable or metastatic) melanoma in adults	Lymphoma-myeloma/455-pembrolizumab-200 mg-monotherapy.pdf	http://www.ncpe.ie/drugs/pembrolizumab-keytruda/	Jun 2016
			Melanoma/pembrolizumab-400 mg-monotherapy-558.pdf		
35	Pembrolizumab (200/400 mg)	Ipilimumab‐refractory patients with unresectable or advanced metastatic melanoma	Lymphoma-myeloma/455-pembrolizumab-200 mg-monotherapy.pdf	Drugs/pembrolizumab-keytruda-for-the-treatment-of-unresectable-or-advanced-metastatic-melanoma-in-adults-refractory-to-ipilimumab/	Jun 2016
			Melanoma/pembrolizumab-400 mg-monotherapy-558.pdf		
36	Ibrutinib	Adults with relapsed or refractory mantle cell lymphoma	Leukemia-bmt/296.pdf	Drugs/ibrutinib-imbruvica-for-mcl/	Aug 2016
37	Ibrutinib	Adults with Waldenström’s macroglobulinaemia who have received at least one prior therapy, or in first line treatment for patients unsuitable for chemo‐immunotherapy	Leukemia-bmt/296.pdf	NA	Aug 2016
38	Ibrutinib	Adults with chronic lymphocytic leukaemia who have received at least one prior therapy, or in first line in the presence of 17p deletion or TP53 mutation in patients unsuitable for chemo‐immunotherapy	Leukemia-bmt/296.pdf	News/ibrutinib-imbruvica/	Aug 2016
39	Ceritinib	Adults with anaplastic lymphoma kinase (ALK)-positive advanced non-small cell lung cancer (NSCLC) previously treated with crizotinib	Lung/340-ceritinib-monotherapy.pdf	Drugs/ceritinib-zykadia/	Dec 2016
40	Ponatinib	Adults with Philadelphia chromosome positive acute lymphoblastic leukaemia (Ph + ALL) who are resistant to dasatinib; who are intolerant to dasatinib and for whom subsequent treatment with imatinib is not clinically appropriate; or who have the T315I mutation	Leukemia-bmt/302.pdf	Drugs/ponatinib-iclusig/	Dec 2016
41	Ponatinib	Adults with chronic phase chronic myeloid leukaemia (CML) who are resistant to dasatinib or nilotinib; who are intolerant to dasatinib or nilotinib and for whom subsequent treatment with imatinib is not clinically appropriate; or who have the T315I mutation	Leukemia-bmt/302.pdf	Drugs/ponatinib-iclusig/	Dec 2016
42	Ponatinib	Adults with accelerated phase chronic myeloid leukaemia (CML) who are resistant to dasatinib or nilotinib; who are intolerant to dasatinib or nilotinib and for whom subsequent treatment with imatinib is not clinically appropriate; or who have the T315I mutation	Leukemia-bmt/302.pdf	Drugs/ponatinib-iclusig/	Dec 2016
43	Ponatinib	Adults with blast phase chronic myeloid leukaemia (CML) who are resistant to dasatinib or nilotinib; who are intolerant to dasatinib or nilotinib and for whom subsequent treatment with imatinib is not clinically appropriate; or who have the T315I mutation	Leukemia-bmt/302.pdf	Drugs/ponatinib-iclusig/	Dec 2016
44	Idelalisib	Monotherapy for adults with follicular lymphoma (FL) that is refractory to two prior lines of treatment	Lymphoma-myeloma/291.pdf	NA	Jan 2017
45	Idelalisib	Combination with riTUXimab for adults with chronic lymphocytic leukaemia (CLL) who have received at least one prior therapy	Leukemia-bmt/389.pdf	Drugs/idelalisib-zydelig/	Jan 2017
46	Idelalisib	Combination with riTUXimab for adults with chronic lymphocytic leukaemia (CLL) as first line treatment in the presence of 17p deletion or TP53 mutation in patients who are not eligible for any other therapies	Leukemia-bmt/389.pdf	Drugs/idelalisib-zydelig/	Jan 2017
47	Idelalisib	Combination with Ofatumumab for adults with chronic lymphocytic leukaemia (CLL) who have received at least one prior therapy	Leukemia-bmt/390.pdf	NA	Jan 2017
48	Idelalisib	Combination with Ofatumumab for adults with chronic lymphocytic leukaemia (CLL) as first line treatment in the presence of 17p deletion or TP53 mutation in patients who are not eligible for any other therapies	Leukemia-bmt/390.pdf	NA	Jan 2017
49	Nintedanib	Combination with docetaxel for adults with locally advanced, metastatic of stage IIIB or IV, or locally recurrent NSCLC of adenocarcinoma tumour histology after first-line chemotherapy	Lung/372.pdf	Drugs/nintedanib-vargatef/	Feb 2017
50	Trifluridine and tipracil	Adults with metastatic colorectal cancer (CRC) who have been previously treated with, or are not considered candidates for, available therapies including fluoropyrimidine-, oxaliplatin- and irinotecan-based chemotherapies, anti-VEGF agents, and anti-EGFR agents	Gastrointestinal/382.pdf	Drugs/trifluridinetipiracil-lonsurf/	Feb 2017
51	Nivolumab 240 mg	Monotherapy for adults with relapsed or refractory classical Hodgkin lymphoma (cHL) after autologous stem cell transplant (ASCT) and treatment with brentuximab vedotin	Genitourinary/483-nivolumab-240 mg-monotherapy-14-day.pdf	Drugs/nivolumab-opdivo-for-classical-hodgkin-lymphoma/	Oct 2017
52	Nivolumab 240/480 mg	As monotherapy for advanced (unresectable or metastatic) melanoma in adults (BRAF positive)	Genitourinary/483-nivolumab-240 mg-monotherapy-14-day.pdf	Drugs/nivolumab-opdivio-for-melanoma/	Oct 2017
			Genitourinary/484-nivolumab-480 mg-monotherapy-28-days.pdf		
53	Nivolumab 240/480 mg	Monotherapy for advanced (unresectable or metastatic) melanoma in adults (BRAF negative)	Genitourinary/483-nivolumab-240 mg-monotherapy-14-day.pdf	Drugs/nivolumab-opdivio-for-melanoma/	Oct 2017
			Genitourinary/484-nivolumab-480 mg-monotherapy-28-days.pdf		
54	Nivolumab 240/480 mg	Monotherapy for advanced renal cell carcinoma (RCC) after prior therapy in adults	Genitourinary/483-nivolumab-240 mg-monotherapy-14-day.pdf	Drugs/nivolumab-opdivo-for-advanced-renal-cell-carcinoma/	Oct 2017
			Genitourinary/484-nivolumab-480 mg-monotherapy-28-days.pdf		
55	Nivolumab Ipilimumab	Combination with ipilimumab for advanced (unresectable or metastatic) melanoma in adults	Melanoma/431-nivolumab-1 mg-ipilimumab-3 mg-therapy.pdf	Drugs/nivolumab-plus-ipilimumab-opdivio-plus-yervoy/	Oct 2017
56	Alectinib	Adults with anaplastic lymphoma kinase (ALK)-positive advanced non-small cell lung cancer (NSCLC) previously treated with crizotinib	Lung/alectinib-monotherapy1.pdf	Drugs/alectinib-alecensa/	Nov 2017
57	Obinutuzumab	Combination with bendamustine for patients with follicular lymphoma (FL) who did not respond or who progressed during or up to 6 months after treatment with rituximab or a rituximab-containing regimen	Lymphoma-myeloma/424-obinutuzumab-and-bendamustine-therapy.pdf	Drugs/obinutuzumab-gazyvaro-for-follicular-lymphoma/	Nov 2017
		Maintenance therapy in patients with follicular lymphoma (FL) who have responded to induction treatment with obinutuzumab and bendamustine or have stable disease	Lymphoma-myeloma/425%20obinutuzumab-maintenance-therapy-following-o-bendamustine-therapy1.pdf		Nov 2017
58	Olaparib	Maintenance treatment of adults with platinum-sensitive relapsed BRCA-mutated (germline and/or somatic)—fallopian tube cancer who are in response (complete response or partial response) to platinum-based chemotherapy	Gynaecology/341-olaparib-monotherapy.pdf	Drugs/olaparib-lynparza-2/	Nov 2017
		Maintenance treatment of adults with platinum-sensitive relapsed BRCA-mutated (germline and/or somatic)—high-grade serous epithelial ovarian cancer who are in response (complete response or partial response) to platinum-based chemotherapy			
		Maintenance treatment of adults with platinum-sensitive relapsed BRCA-mutated (germline and/or somatic)—primary peritoneal cancer who are in response (complete response or partial response) to platinum-based chemotherapy			
59	Vismodegib	Adults with local advanced basal cell carcinoma inappropriate for surgery or radiotherapy	Melanoma/vismodegib-monotherapy.pdf	Drugs/vismodegib-erivedge/	Nov 2017
60	Vismodegib	Adults with symptomatic metastatic basal cell carcinoma (MBCC)	Melanoma/vismodegib-monotherapy.pdf	Drugs/vismodegib-erivedge/	Nov 2017
61	Cobimetinib	Combination with vemurafenib for adults with unresectable or metastatic melanoma with a BRAF V600 mutation	Sarcoma/mifamurtide.pdf	Drugs/cobimetinib-cotellic/	Apr 2018
62	Daratumumab	Monotherapy for adults with relapsed and refractory multiple myeloma whose prior therapy included a proteasome inhibitor and an immunomodulatory agent and who have demonstrated disease progression on the last therapy	Lymphoma-myeloma/daratumumab monotherapy.pdf	Drugs/daratumumab-darzalex/	Apr 2018
63	Pembrolizumab (200 mg/400 mg)	First-line for metastatic non-small cell lung carcinoma (NSCLC) in adults whose tumours express PD-L1 with a ≥ 50% tumour proportion score (TPS) with no EGFR or ALK positive tumour mutations	Lymphoma-myeloma/455-pembrolizumab-200 mg-monotherapy.pdf	Drugs/pembrolizumab-keytruda-for-first-line-nsclc/	Apr 2018
			Melanoma/pembrolizumab-400 mg-monotherapy-558.pdf		
64	Trametinib	Combination with dabrafenib for adults with unresectable or metastatic melanoma with a BRAF V600 mutation	Melanoma/dabrafenib and trametinib therapy.pdf	Drugs/trametinib-mekinist/	Apr 2018
65	Nivolumab	Monotherapy for squamous cell cancer of the head and neck in adults progressing on or after platinum-based therapy	Headandneck/483-nivolumab-240 mg-monotherapy.pdf	Drugs/nivolumab-opdivo-for-head-and-neck-cancer/	May 2018
66	Palbociclib	Hormone receptor (HR)-positive, human epidermal growth factor receptor 2 (HER2)-negative locally advanced or metastatic breast cancer in combination with fulvestrant in women who have received prior endocrine therapy (2nd line)	Breast/414-palbociclib-therapy-28-days.pdf	Drugs/Palbociclib-Ibrance/	Jun 2018
67	Palbociclib	Hormone receptor (HR)-positive, human epidermal growth factor receptor 2 (HER2)-negative locally advanced or metastatic breast cancer in combination with an aromatase inhibitor (1st line)	Breast/414-palbociclib-therapy-28-days.pdf	Drugs/palbociclib-ibrance/	Jun 2018
68	Carfilzomib	Carlfilzomib, lenalidomide and dexamethasone for adults with multiple myeloma who have received at least one pror therapy	Lymphoma-myeloma/405-carfilzomib-lenalidomide-and-dexamethasone-krd-therapy-28-day.pdf	Drugs/carfilzomib-kyprolis/	Sep 2018
69	Nivolumab	Monotherapy for locally advanced or metastatic non-small cell lung cancer (NSCLC) after prior chemotherapy in adults	Headandneck/483-nivolumab-240 mg-monotherapy.pdf	Drugs/nivolumab-opdivio-for-non-squamous-nsclc/	Sep 2018
70	Pembrolizumab (200 mg/400 mg)	Monotherapy for adults with relapsed or refractory classical Hodgkin lymphoma (cHL) who are transplant-ineligible and have failed brentuximab vedotin	Lymphoma-myeloma/455-pembrolizumab-200 mg-monotherapy.pdf	Drugs/pembrolizumab-keytruda-for-classical-hodgkin-lymphoma/	Nov 2018
			Melanoma/pembrolizumab-400 mg-monotherapy-558.pdf		
71	Ixazomib	Combination with lenalidomide and dexamethasone for adults with multiple myeloma who have received at least one prior therapy	Lymphoma-myeloma/516-ixazomib-lenalidomide-and-dexamethasone-therapy-28-day.pdf	Drugs/ixazomib-ninlaro/	Dec 2018
72	Venetoclax	Chronic lymphocytic leukaemia in the absence of 17p deletion or TP53 mutation in adults who have failed both chemoimmunotherapy and a B-cell receptor pathway inhibitor	Leukemia-bmt/400.pdf	Drugs/venetoclax-venclyxto/	Dec 2018
73	Venetoclax	Chronic lymphocytic leukaemia in the presence of 17p deletion or TP53 mutation in adults who are unsuitable for or have failed a B-cell receptor pathway inhibitor	Leukemia-bmt/400.pdf	Drugs/venetoclax-venclyxto/	Dec 2018
74	Cabozantinib	Advanced renal cell carcinoma in adults following prior VEGF targeted therapy	Genitourinary/518 cabozantinib therapy.pdf	Drugs/cabozantinib-cabometyx/	Jan 2019
75	Ribociclib	Postmenopausal women with hormone receptor (HR) positive, human epidermal growth factor receptor 2 (HER2) negative locally advanced or metastatic breast cancer as initial endocrine-based therapy in combination with an aromatase inhibitor	Breast/525-ribociclib-therapy-28-day.pdf	Drugs/ribociclib-kisqali/	Feb 2019
76	Atezolizumab	Adults with locally advanced or metastatic non-small cell lung cancer after prior chemotherapy	Lung/544-atezolizumab-1200 mg-Monotherapy.pdf	Drugs/atezolizumab-tecentriq/	Mar 2019
77	Avelumab	Adults with metastatic Merkel cell carcinoma who have received 1 or more lines of chemotherapy for metastatic disease (1st line)	Melanoma/535.pdf	Drugs/avelumab-bavencio/	May 2019
78	Avelumab	Adult with metastatic Merkel cell carcinoma who have received 1 or more lines of chemotherapy for metastatic disease (2nd line)	Melanoma/535.pdf	Drugs/avelumab-bavencio/	May 2019
79	Blinatumomab	Adults with relapsed or refractory B cell precursor (BCP) Philadelphia chromosome negative acute lymphoblastic leukaemia (ALL) who have received no prior salvage treatment for relapsed/refractory disease and are considered eligible for transplant	Leukemia-bmt/538-blinatumomab-therapy.pdf	Drugs/blinatumomab-blincyto/	May 2019
80	Blinatumomab	Paediatric patients aged 1 year and older with Philadelphia chromosome negative B-cell precursor ALL which is refractory or in relapse after receiving at least two prior therapies or in relapse after receiving prior allogeneic hematopoietic stem cell transplantation	p567-blinatumomab-paediatric-therapy.pdf	Drugs/blinatumomab-blincyto-paediatric-all/	May 2019
81	Encorafenib and Binimetinib	Adults with advanced (unresectable or metastatic) melanoma with a BRAF V600 mutation	Melanoma/563-envorafenib-and-binimetinib-therapy.pdf	Drugs/encorafenib-braftovi-binimetinib-mektovi/	May 2019
82	Inotuzumab	Monotherapy for adults with relapsed or refractory CD22-positive B cell precursor acute lymphoblastic leukaemia (ALL). Adults with Philadelphia chromosome positive (Ph+) relapsed or refractory B cell precursor ALL should have failed treatment with at least 1 tyrosine kinase inhibitor (TKI)	Leukemia-bmt/537-inotuzumab-ozogamicin-monotherapy.pdf	Drugs/inotuzumab-ozogamicin-besponsa/	May 2019
83	Obinutuzumab	Combination with chemotherapy, followed by maintenance treatment in patients achieving a response, for previously untreated advanced follicular lymphoma (FL)	Lymphoma-myeloma/549-obinutuzumab-and-chop-therapy-%E2%80%93–21-day.pdf	Drugs/obinutuzumab-gazyvaro-for-previously-untreated-advanced-follicular-lymphoma/	May 2019
84	Alectinib	As monotherapy is indicated for the first-line treatment of adults with anaplastic lymphoma kinase (ALK)-positive advanced non-small cell lung cancer (NSCLC)	Lung/alectinib-monotherapy1.pdf	Drugs/alectinib-alecensa/	Jun 2019
85	Brigatinib	Adults with anaplastic lymphoma kinase (ALK) positive advanced non-small cell lung cancer (NSCLC) previously treated with crizotinib	Lung/562-brigatinib-therapy.pdf	Drugs/brigatinib-alunbrig/	Jun 2019
86	Lorlatinib	Monotherapy for adults with anaplastic lymphoma kinase (ALK)-positive advanced non-small cell lung cancer (NSCLC), following disease progression on (i) alectinib or ceritinib as the first ALK-targeted treatment or (ii) crizotinib and at least one other ALK-targeted treatment	Lung/570 lorlatinib therapy.pdf	Drugs/lorlatinib-lorviqua/	Oct 2019
87	Tivozanib	First line for adults with advanced renal cell carcinoma (RCC) and for adults who are VEGFR and mTOR pathway inhibitor-naïve following disease progression after one prior treatment with cytokine therapy for advanced RCC	Genitourinary/564-tivozanib-therapy.pdf	Drugs/tivozanib-fotivda/	Oct 2019
88	Dacomitinib	Dacomitinib (Vizimpro®) as monotherapy, for the first-line treatment of adults with locally advanced or metastatic non-small cell lung cancer (NSCLC) with epidermal growth factor receptor (EGFR)-activating mutations	Lung/565-dacomitinib-monotherapy.pdf	Drugs/dacomitinib-vizimpro/	Nov 2019
89	Osimertinib	Adults with locally advanced or metastatic epidermal growth factor receptor (EGFR) T790M mutation-positive non-small cell lung cancer (NSCLC)	Lung/353-Osimertinib-Monotherapy.Pdf	Drugs/osimertinib-tagrisso-for-the-first-line-treatment-of-metastatic-nsclc/	Jul 2020
90	Pertuzumab	Pertuzumab in combination with trastuzumab and chemotherapy for the neoadjuvant treatment of adults with HER2-positive, locally advanced, inflammatory, or early stage breast cancer at high risk of recurrence	Breast/350%20pertuzumab%20and%20trastuzumab%20and%20chemotherapy.pdf	Drugs/pertuzumab-perjeta-for-her2-positive-breast-cancer/	Jul 2020
91	Venetoclax	Combination with rituximab for adults with chronic lymphocytic leukaemia (CLL) who have received at least one prior therapy	Leukemia-bmt/575-venetoclax-and-rituximab-therapy.pdf	Drugs/venetoclax-venclyxto-in-combination-with-rituximab/	Jul 2020

^a^NCCP URL stem: https://www.hse.ie/eng/services/list/5/cancer/profinfo/chemoprotocols

^b^NCPE URL stem: http://www.ncpe.ie/

**Table 5:** Drug and disease information

Pair number	Drug name	Proprietary name	Market authorisation holder	Indication	ICD 10 code	Disease category	Metastatic disease	Mechanism of action	Orphan status
1	Ipilimumab	Yervoy	Bristol-Myers Squibb	Adults with advanced (unresectable or metastatic) malignant melanoma	C43	Skin	Yes	Monoclonal antibodies	Negative
2	Abiraterone	Zytiga	Janssen-Cilag	Metastatic castration resistant prostate cancer which has progressed on or after a docetaxel-based chemotherapy regimen	C61	Prostate	Yes	Immunomodulating agents and other non-antineoplastic therapies	Negative
3	Tegafur/ Gimeracil/ Oteracil	Teysuno	Nordic Group	Advanced gastric cancer in combination with cisplatin	C16	Digestive Organs	Yes	Antimetabolites, plant alkaloids, cytotoxic antibiotic and related substances	Negative
4	Axitinib	Inlyta	Pfizer	Adults with advanced RCC after failure, on a previous line of therapy, i.e., treatment with SUNItinib, or a cytokine	C64	Kidney	No	Protein kinase inhibitors	Negative, Withdrawn
5	Cabazitaxel	Jevtana	Sanofi-Aventis	Metastatic castration resistant prostate cancer previously treated with docetaxel containing regimen	C61	Prostate	Yes	Antimetabolites, plant alkaloids, cytotoxic antibiotic and related substances	Negative
6	Mifamurtide	Mepact	Takeda	High-grade resectable non-metastatic osteosarcoma after macroscopically complete surgical resection, in children, adolescents and young adults	C41	Other	No	Immunomodulating agents and other non-antineoplastic therapies	Negative
7	Vemurafenib	Zelboraf	Roche	Adults with BRAF V600 mutation-positive unresectable or metastatic melanoma	C43	Skin	Yes	Protein kinase inhibitors	Negative
8	Afatinib	Gilotrif	Boehringer Ingelheim	As monotherapy for EGFR TKI-naïve adults with locally advanced or metastatic non-small cell lung cancer (NSCLC) with activating EGFR mutation(s)	C34	Lung	Yes	Protein kinase inhibitors	Negative
9	Bosutinib	Bosulif	Pfizer	Adults with chronic phase, accelerated phase, and blast phase Ph + CML previously treated with one or more TKI(s) and for whom imatinib, nilotinib and dasatinib are not considered appropriate treatment options	C92	Leukaemias	No	Protein kinase inhibitors	Positive, 1/13/818
10	Decitabine	Dacogen	Janssen-Cilag	Adults aged 65 years and above with newly diagnosed de novo or secondary AML, according to the WHO classification, who are not candidates for standard induction chemotherapy	C92	Leukaemias	No	Antimetabolites, plant alkaloids, cytotoxic antibiotic and related substances	Positive, 3/06/370
11	Eribulin	Halaven	Eisai	LABC or MBC which has progressed after at least two chemotherapeutic regimens for advanced disease. Prior therapy should have included an anthracycline and a taxane unless patients were not suitable for these treatments	C50	Breast	Yes	Other antineoplastic agents	Negative
12	Pertuzumab	Perjeta	Roche	Adults with HER2-positive MBC or locally recurrent unresectable breast cancer, who have not received previous anti-HER2 therapy or chemotherapy for their metastatic disease	C50	Breast	Yes	Monoclonal antibodies	Negative
13	Ruxolitinib	Jakavi	Novartis	Disease-related splenomegaly or symptoms in adults with post polycythaemia vera myelofibrosis	D45	Other	No	Protein kinase inhibitors	Negative
				Disease-related splenomegaly or symptoms in adults with primary myelofibrosis (chronic idiopathic myelofibrosis)	D47				
				Disease-related splenomegaly or symptoms in adults with post essential thrombocythaemia myelofibrosis	D47				
14	Aflibercept	Eylea	Bayer	Combination with irinotecan/5-fluorouracil/folinic acid (FOLFIRI) chemotherapy in adults with metastatic colorectal cancer that is resistant to or has progressed after an oxoliplatin-containing regimen	C18	Digestive Organs	Yes	Immunomodulating agents and other non-antineoplastic therapies	Negative
15	Crizotinib	Xalkori	Pfizer	Adults with previously treated anaplastic lymphoma kinase (ALK)-positive advanced non-small cell lung cancer (NSCLC)	C34	Lung	No	Protein kinase inhibitors	Negative
16	Vandetanib	Caprelsa	Genzyme	Aggressive and symptomatic medullary thyroid cancer (MTC) in patients with unresectable locally advanced or metastatic disease	C73	Other	Yes	Protein kinase inhibitors	Negative
17	Brentuximab vedotin	Adcetris	Takeda	Adults with relapsed or refractory CD30 + Hodgkin lymphoma (HL): following autologous stem cell transplant (ASCT)	C81	Lymphomas	No	Monoclonal antibodies	Negative
18	Brentuximab vedotin	Adcetris	Takeda	Adults with relapsed or refractory CD30 + Hodgkin lymphoma (HL): following at least two prior therapies when ASCT or multi-agent chemotherapy is not an option	C81	Lymphomas	No	Monoclonal antibodies	Negative
19	Brentuximab vedotin	Adcetris	Takeda	Adults with relapsed or refractory systemic anaplastic large cell lymphoma (sALCL)	C84	Lymphomas	No	Monoclonal antibodies	Negative
20	Enzalutamide	Xtandi	Astellas	Adults with metastatic castration-resistant prostate cancer whose disease has progressed on or after docetaxel	C61	Prostate	Yes	Immunomodulating agents and other non-antineoplastic therapies	Negative
21	Dabrafenib	Tafinlar	Novartis	Adults with unresectable or metastatic melanoma with the BRAF V600 mutation	C43	Skin	Yes	Protein kinase inhibitors	Negative
22	Regorafenib	Stivarga	Bayer	Adults unresectable or metastatic gastrointestinal stromal tumours (GIST) who progressed on or are intolerant to prior treatment with imatinib and sunitinib	C26	Digestive Organs	Yes	Protein kinase inhibitors	Negative
23	Regorafenib	Stivarga	Bayer	Adults with metastatic colorectal cancer (mCRC) who have been previously treated with, or are not considered candidates for, available therapies. These include fluoropyrimidine-based chemotherapy, anti-VEGF and anti-EGFR therapies	C18	Digestive Organs	Yes	Protein kinase inhibitors	Negative
24	Abiraterone	Zytiga	Janssen-Cilag	Metastatic castration resistant prostate cancer in men who are asymptomatic or mildly symptomatic after failure of androgen deprivation therapy in whom chemotherapy is not yet clinically indicated	C61	Prostate	Yes	Immunomodulating agents and other non-antineoplastic therapies	Negative
25	Radium 223	Xofigo	Bayer	Adults with castration-resistant prostate cancer, symptomatic bone metastases and no known visceral metastases	C61	Prostate	Yes	Immunomodulating agents and other non-antineoplastic therapies	Negative
26	Obinutuzumab	Gazyvaro	Roche	Combination with chlorambucil for adults with previously untreated chronic lymphocytic leukaemia (CLL) and with comorbidities making them unsuitable for full-dose fludarabine based therapy	C91	Leukaemias	No	Monoclonal antibodies	Negative
27	Pixantrone	Pixuvri	Les Laboratoires Servier	Monotherapy for adults with multiply relapsed or refractory aggressive Non-Hodgkin B-cell Lymphomas (NHL)	C85	Other blood cancers	No	Antimetabolites, plant alkaloids, cytotoxic antibiotic and related substances	Negative, Withdrawn
28	Siltuximab	Pixuvri	Les Laboratoires Servier	Adults with Multicentric Castleman’s disease (MCD) who are human immunodeficiency virus (HIV) negative and human herpes virus 8 (HHV-8) negative	D36	Other	Yes	Immunomodulating agents and other non-antineoplastic therapies	Negative
29	Trastuzumab Emtansine	Kadcyla	Roche	Adults with HER2-positive, unresectable locally advanced or metastatic breast cancer who previously received trastuzumab and a taxane, separately or in combination. Patients should have either: received prior therapy for locally advanced or metastatic disease, or developed disease recurrence during or within 6 months of completing adjuvant therapy	C50	Breast	Yes	Monoclonal antibodies	Negative
30	Enzalutamide	Xtandi	Astellas	Metastatic castrate resistant prostate cancer in men who are asymptomatic or mildly symptomatic after failure of androgen deprivation therapy (ADT) in whom chemotherapy is not yet clinically indicated	C61	Prostate	Yes	Immunomodulating agents and other non-antineoplastic therapies	Negative
31	Lenvatinib	Lenvima	Eisai	Adults with progressive, locally advanced or metastatic, differentiated (papillary/follicular/Hürthle cell) thyroid carcinoma (DTC), refractory to radioactive iodine	C73	Other	Yes	Protein kinase inhibitors	Negative, Withdrawn
32	Nab-Paclitaxel	Abraxane	Celgene	Combination with gemcitabine for the first-line treatment of adults with metastatic adenocarcinoma of the pancreas	C25	Digestive Organs	Yes	Antimetabolites, plant alkaloids, cytotoxic antibiotic and related substances	Positive, 3/06/419
33	Pomalidomide	Imnovid	Celgene	Combination with dexamethasone for adults with relapsed and refractory multiple myeloma who have received at least two prior treatment regimens, including both lenalidomide and bortezomib, and have demonstrated disease progression on the last therapy	C90	Other blood cancers	No	Immunomodulating agents and other non-antineoplastic therapies	Negative
34	Pembrolizumab (200/400 mg)	Keytruda	Merck Sharp & Dohme	First line monotherapy for advanced (unresectable or metastatic) melanoma in adults	C43	Skin	Yes	Monoclonal antibodies	Negative
35	Pembrolizumab (200/400 mg)	Keytruda	Merck Sharp & Dohme	Ipilimumab‐refractory patients with unresectable or advanced metastatic melanoma	C43	Skin	Yes	Monoclonal antibodies	Negative
36	Ibrutinib	Imbruvica	Janssen-Cilag	Adults with relapsed or refractory mantle cell lymphoma	C83	Lymphomas	No	Protein kinase inhibitors	Positive, 3/13/1115
37	Ibrutinib	Imbruvica	Janssen-Cilag	Adults with Waldenström’s macroglobulinaemia who have received at least one prior therapy, or in first line treatment for patients unsuitable for chemo‐immunotherapy	C88	Other blood cancers	No	Protein kinase inhibitors	Positive, 3/14/1264
38	Ibrutinib	Imbruvica	Janssen-Cilag	Adults with chronic lymphocytic leukaemia who have received at least one prior therapy, or in first line in the presence of 17p deletion or TP53 mutation in patients unsuitable for chemo‐immunotherapy	C91	Leukaemias	No	Protein kinase inhibitors	Positive, 3/14/1264
39	Ceritinib	Zykadia	Novartis	Adults with anaplastic lymphoma kinase (ALK)-positive advanced non-small cell lung cancer (NSCLC) previously treated with crizotinib	C34	Lung	No	Protein kinase inhibitors	Negative
40	Ponatinib	Iclusig	Incyte	Adults with Philadelphia chromosome positive acute lymphoblastic leukaemia (Ph + ALL) who are resistant to dasatinib; who are intolerant to dasatinib and for whom subsequent treatment with imatinib is not clinically appropriate; or who have the T315I mutation	C91	Leukaemias	Yes	Protein kinase inhibitors	Negative
41	Ponatinib	Iclusig	Incyte	Adults with chronic phase chronic myeloid leukaemia (CML) who are resistant to dasatinib or nilotinib; who are intolerant to dasatinib or nilotinib and for whom subsequent treatment with imatinib is not clinically appropriate; or who have the T315I mutation	C92	Leukaemias	Yes	Protein kinase inhibitors	Negative
42	Ponatinib	Iclusig	Incyte	Adults with accelerated phase chronic myeloid leukaemia (CML) who are resistant to dasatinib or nilotinib; who are intolerant to dasatinib or nilotinib and for whom subsequent treatment with imatinib is not clinically appropriate; or who have the T315I mutation	C92	Leukaemias	Yes	Protein kinase inhibitors	Negative
43	Ponatinib	Iclusig	Incyte	Adults with blast phase chronic myeloid leukaemia (CML) who are resistant to dasatinib or nilotinib; who are intolerant to dasatinib or nilotinib and for whom subsequent treatment with imatinib is not clinically appropriate; or who have the T315I mutation	C92	Leukaemias	Yes	Protein kinase inhibitors	Negative
44	Idelalisib	Zydelig	Gilead	Monotherapy for adults with follicular lymphoma (FL) that is refractory to two prior lines of treatment	C82	Lymphomas	No	Other antineoplastic agents	Negative, Withdrawn
45	Idelalisib	Zydelig	Gilead	Combination with riTUXimab for adults with chronic lymphocytic leukaemia (CLL) who have received at least one prior therapy	C91	Leukaemias	No	Other antineoplastic agents	Negative, Withdrawn
46	Idelalisib	Zydelig	Gilead	Combination with riTUXimab for adults with chronic lymphocytic leukaemia (CLL) as first line treatment in the presence of 17p deletion or TP53 mutation in patients who are not eligible for any other therapies	C91	Leukaemias	No	Other antineoplastic agents	Negative, Withdrawn
47	Idelalisib	Zydelig	Gilead	Combination with Ofatumumab for adults with chronic lymphocytic leukaemia (CLL) who have received at least one prior therapy	C91	Leukaemias	No	Other antineoplastic agents	Negative, Withdrawn
48	Idelalisib	Zydelig	Gilead	Combination with Ofatumumab for adults with chronic lymphocytic leukaemia (CLL) as first line treatment in the presence of 17p deletion or TP53 mutation in patients who are not eligible for any other therapies	C91	Leukaemias	No	Other antineoplastic agents	Negative, Withdrawn
49	Nintedanib	Ofev	Boehringer Ingelheim	Combination with docetaxel for adults with locally advanced, metastatic of stage IIIB or IV, or locally recurrent NSCLC of adenocarcinoma tumour histology after first-line chemotherapy	C34	Lung	Yes	Protein kinase inhibitors	Negative
50	Trifluridine and Tipracil	Lonsurf	Les Laboratoires Servier	Adults with metastatic colorectal cancer (CRC) who have been previously treated with, or are not considered candidates for, available therapies including fluoropyrimidine-, oxaliplatin- and irinotecan-based chemotherapies, anti-VEGF agents, and anti-EGFR agents	C18	Digestive Organs	Yes	Antimetabolites, plant alkaloids, cytotoxic antibiotic and related substances	Negative
51	Nivolumab 240 mg	Opdivo	Bristol-Myers Squibb	Monotherapy for adults with relapsed or refractory classical Hodgkin lymphoma (cHL) after autologous stem cell transplant (ASCT) and treatment with brentuximab vedotin	C81	Lymphomas	No	Monoclonal antibodies	Negative
52	Nivolumab 240/480 mg	Opdivo	Bristol-Myers Squibb	As monotherapy for advanced (unresectable or metastatic) melanoma in adults (BRAF positive)	C43	Skin	Yes	Monoclonal antibodies	Negative
53	Nivolumab 240/480 mg	Opdivo	Bristol-Myers Squibb	Monotherapy for advanced (unresectable or metastatic) melanoma in adults (BRAF negative)	C43	Skin	Yes	Monoclonal antibodies	Negative
54	Nivolumab 240/480 mg	Opdivo	Bristol-Myers Squibb	Monotherapy for advanced renal cell carcinoma (RCC) after prior therapy in adults	C64	Kidney	Yes	Monoclonal antibodies	Negative
55	Nivolumab Ipilimumab	Opdivo	Bristol-Myers Squibb	Combination with ipilimumab for advanced (unresectable or metastatic) melanoma in adults	C43	Skin	Yes	Monoclonal antibodies	Negative
56	Alectinib	Alecensa	Roche	Adults with anaplastic lymphoma kinase (ALK)-positive advanced non-small cell lung cancer (NSCLC) previously treated with crizotinib	C34	Lung	No	Protein kinase inhibitors	Negative
57	Obinutuzumab	Gazyvaro	Roche	Combination with bendamustine for patients with follicular lymphoma (FL) who did not respond or who progressed during or up to 6 months after treatment with rituximab or a rituximab-containing regimen	C82	Lymphomas	No	Monoclonal antibodies	Positive, 3/15/1504
			Roche	Maintenance therapy in patients with follicular lymphoma (FL) who have responded to induction treatment with obinutuzumab and bendamustine or have stable disease	C82	Lymphomas	No	Monoclonal antibodies	Positive, 3/15/1504
58	Olaparib	Lynparza	AstraZeneca	Maintenance treatment of adults with platinum-sensitive relapsed BRCA-mutated (germline and/or somatic)—fallopian tube cancer who are in response (complete response or partial response) to platinum-based chemotherapy	C48	Other	No	Other antineoplastic agents	Negative
			AstraZeneca	Maintenance treatment of adults with platinum-sensitive relapsed BRCA-mutated (germline and/or somatic)—high-grade serous epithelial ovarian cancer who are in response (complete response or partial response) to platinum-based chemotherapy	C56	Other	No	Other antineoplastic agents	Negative
			AstraZeneca	Maintenance treatment of adults with platinum-sensitive relapsed BRCA-mutated (germline and/or somatic)—primary peritoneal cancer who are in response (complete response or partial response) to platinum-based chemotherapy	C57	Other	No	Other antineoplastic agents	Negative
59	Vismodegib	Erivedge	Roche	Adults with local advanced basal cell carcinoma inappropriate for surgery or radiotherapy	C44	Skin	No	Other antineoplastic agents	Negative
60	Vismodegib	Erivedge	Roche	Adults with symptomatic metastatic basal cell carcinoma (MBCC)	C44	Skin	Yes	Other antineoplastic agents	Negative
61	Cobimetinib	Cotellic	Roche	Combination with vemurafenib for adults with unresectable or metastatic melanoma with a BRAF V600 mutation	C43	Skin	Yes	Protein kinase inhibitors	Negative
62	Daratumumab	Darzalex	Janssen-Cilag	Monotherapy for adults with relapsed and refractory multiple myeloma whose prior therapy included a proteasome inhibitor and an immunomodulatory agent and who have demonstrated disease progression on the last therapy	C90	Other blood cancers	No	Monoclonal antibodies	Negative
63	Pembrolizumab 200 mg/400 mg	Keytruda	Merck Sharp & Dohme	First-line for metastatic non-small cell lung carcinoma (NSCLC) in adults whose tumours express PD-L1 with a ≥ 50% tumour proportion score (TPS) with no EGFR or ALK positive tumour mutations	C34	Lung	Yes	Monoclonal antibodies	Negative
64	Trametinib	Mekinist	Novartis	Combination with dabrafenib for adults with unresectable or metastatic melanoma with a BRAF V600 mutation	C43	Skin	Yes	Protein kinase inhibitors	Negative
65	Nivolumab	Opdivo	Bristol-Myers Squibb	Monotherapy for squamous cell cancer of the head and neck in adults progressing on or after platinum-based therapy	C76	Other	No	Monoclonal antibodies	Negative
66	Palbociclib	Ibrance	Pfizer	Hormone receptor (HR)-positive, human epidermal growth factor receptor 2 (HER2)-negative locally advanced or metastatic breast cancer in combination with fulvestrant in women who have received prior endocrine therapy (2nd line)	C50	Breast	Yes	Protein kinase inhibitors	Negative
67	Palbociclib	Ibrance	Pfizer	Hormone receptor (HR)-positive, human epidermal growth factor receptor 2 (HER2)-negative locally advanced or metastatic breast cancer in combination with an aromatase inhibitor (1st line)	C50	Breast	Yes	Protein kinase inhibitors	Negative
68	Carfilzomib	Kyprolis	Amgen	Carlfilzomib, lenalidomide and dexamethasone for adults with multiple myeloma who have received at least one pror therapy	C90	Other blood cancers	No	Other antineoplastic agents	Positive, 3/08/548
69	Nivolumab	Opdivo	Bristol-Myers Squibb	Monotherapy for locally advanced or metastatic non-small cell lung cancer (NSCLC) after prior chemotherapy in adults	C34	Lung	Yes	Monoclonal antibodies	Negative
70	Pembrolizumab 200 mg/400 mg	Keytruda	Merck Sharp & Dohme	Monotherapy for adults with relapsed or refractory classical Hodgkin lymphoma (cHL) who are transplant-ineligible and have failed brentuximab vedotin	C81	Lymphomas	No	Monoclonal antibodies	Negative
71	Ixazomib	Ninlaro	Takeda	Combination with lenalidomide and dexamethasone for adults with multiple myeloma who have received at least one prior therapy	C90	Other blood cancers	No	Other antineoplastic agents	Negative
72	Venetoclax	Venclyxto	AbbVie	Chronic lymphocytic leukaemia in the absence of 17p deletion or TP53 mutation in adults who have failed both chemoimmunotherapy and a B-cell receptor pathway inhibitor	C91	Leukaemias	No	Other antineoplastic agents	Negative
73	Venetoclax	Venclyxto	AbbVie	Chronic lymphocytic leukaemia in the presence of 17p deletion or TP53 mutation in adults who are unsuitable for or have failed a B-cell receptor pathway inhibitor	C91	Leukaemias	No	Other antineoplastic agents	Negative
74	Cabozantinib	Cometriq	Ipsen	Advanced renal cell carcinoma in adults following prior VEGF targeted therapy	C64	Kidney	Yes	Protein kinase inhibitors	Negative
75	Ribociclib	Kisqali	Novartis	Postmenopausal women with hormone receptor (HR) positive, human epidermal growth factor receptor 2 (HER2) negative locally advanced or metastatic breast cancer as initial endocrine-based therapy in combination with an aromatase inhibitor	C50	Breast	Yes	Protein kinase inhibitors	Negative
76	Atezolizumab	Tecentriq	Roche	Adults with locally advanced or metastatic non-small cell lung cancer after prior chemotherapy	C34	Lung	Yes	Monoclonal antibodies	Negative
77	Avelumab	Bavencio	Merck	Adults with metastatic Merkel cell carcinoma who have received 1 or more lines of chemotherapy for metastatic disease (1st line)	C4A	Skin	Yes	Monoclonal antibodies	Negative
78	Avelumab	Bavencio	Merck	Adult with metastatic Merkel cell carcinoma who have received 1 or more lines of chemotherapy for metastatic disease (2nd line)	C4A	Skin	Yes	Monoclonal antibodies	Negative
79	Blinatumomab	Blincyto	Amgen	Adults with relapsed or refractory B cell precursor (BCP) Philadelphia chromosome negative acute lymphoblastic leukaemia (ALL) who have received no prior salvage treatment for relapsed/refractory disease and are considered eligible for transplant	C91	Leukaemias	No	Monoclonal antibodies	Positive, 1/15/1047
80	Blinatumomab	Blincyto	Amgen	Paediatric patients aged 1 year and older with Philadelphia chromosome negative B-cell precursor ALL which is refractory or in relapse after receiving at least two prior therapies or in relapse after receiving prior allogeneic hematopoietic stem cell transplantation	C91	Leukaemias	No	Monoclonal antibodies	Positive, 1/15/1048
81	Encorafenib and Binimetinib	Braftovi	Pierre Fabre Médicament	Adults with advanced (unresectable or metastatic) melanoma with a BRAF V600 mutation	C43	Skin	Yes	Protein kinase inhibitors	Negative
82	Inotuzumab	Besponsa	Pfizer	Monotherapy for adults with relapsed or refractory CD22-positive B cell precursor acute lymphoblastic leukaemia (ALL). Adults with Philadelphia chromosome positive (Ph+) relapsed or refractory B cell precursor ALL should have failed treatment with at least 1 tyrosine kinase inhibitor (TKI)	C91	Leukaemias	No	Monoclonal antibodies	Negative
83	Obinutuzumab	Gazyvaro	Roche	Combination with chemotherapy, followed by maintenance treatment in patients achieving a response, for previously untreated advanced follicular lymphoma (FL)	C82	Lymphomas	No	Monoclonal antibodies	Positive, 3/15/1504
84	Alectinib	Alecensa	Roche	As monotherapy is indicated for the first-line treatment of adults with anaplastic lymphoma kinase (ALK)-positive advanced non-small cell lung cancer (NSCLC)	C34	Lung	No	Protein kinase inhibitors	Negative
85	Brigatinib	Alunbrig	Takeda	Adults with anaplastic lymphoma kinase (ALK) positive advanced non-small cell lung cancer (NSCLC) previously treated with crizotinib	C34	Lung	No	Protein kinase inhibitors	Negative
86	Lorlatinib	Lorviqua	Pfizer	Monotherapy for adults with anaplastic lymphoma kinase (ALK)-positive advanced non-small cell lung cancer (NSCLC), following disease progression on (i) alectinib or ceritinib as the first ALK-targeted treatment or (ii) crizotinib and at least one other ALK-targeted treatment	C34	Lung	No	Protein kinase inhibitors	Negative
87	Tivozanib	Fotivda	EUSA Pharma	First line for adults with advanced renal cell carcinoma (RCC) and for adults who are VEGFR and mTOR pathway inhibitor-naïve following disease progression after one prior treatment with cytokine therapy for advanced RCC	C64	Kidney	No	Protein kinase inhibitors	Negative
88	Dacomitinib	Darzalex	Janssen-Cilag	Dacomitinib (Vizimpro®) as monotherapy, for the first-line treatment of adults with locally advanced or metastatic non-small cell lung cancer (NSCLC) with epidermal growth factor receptor (EGFR)-activating mutations	C34	Lung	Yes	Protein kinase inhibitors	Negative
89	Osimertinib	Tagrisso	AstraZeneca	Adults with locally advanced or metastatic epidermal growth factor receptor (EGFR) T790M mutation-positive non-small cell lung cancer (NSCLC)	C34	Lung	Yes	Protein kinase inhibitors	Negative
90	Pertuzumab	Perjeta	Roche	Pertuzumab in combination with trastuzumab and chemotherapy for the neoadjuvant treatment of adults with HER2-positive, locally advanced, inflammatory, or early stage breast cancer at high risk of recurrence	C50	Breast	No	Monoclonal antibodies	Negative
91	Venetoclax	Venclyxto	AbbVie	Combination with rituximab for adults with chronic lymphocytic leukaemia (CLL) who have received at least one prior therapy	C91	Leukaemias	No	Other antineoplastic agents	Negative

**Table 6:** Reimbursement process

Pair number	Drug name	Indication	Appraisal pathway	Funding stream	Time taken to reimburse, months	HTA summary length, pages	Subsequent price negotiations
1	Ipilimumab	Adults with advanced (unresectable or metastatic) malignant melanoma	After HTA	ODMS	11	3	Yes
2	Abiraterone	Metastatic castration resistant prostate cancer which has progressed on or after a docetaxel-based chemotherapy regimen	After HTA	PCRS	7	5	Yes
3	Tegafur/Gimeracil /Oteracil	Advanced gastric cancer in combination with cisplatin	At RR	PCRS	10	N/A	No
4	Axitinib	Adults with advanced RCC after failure, on a previous line of therapy, i.e., treatment with SUNItinib, or a cytokine	Undocumented	PCRS	3	N/A	No
5	Cabazitaxel	Metastatic castration resistant prostate cancer previously treated with docetaxel containing regimen	After HTA	ODMS	21	5	Yes
6	Mifamurtide	High-grade resectable non-metastatic osteosarcoma after macroscopically complete surgical resection, in children, adolescents and young adults	Undocumented	ODMS	32	N/A	No
7	Vemurafenib	Adults with BRAF V600 mutation-positive unresectable or metastatic melanoma	After HTA	PCRS	14	3	Yes
8	Afatinib	As monotherapy for EGFR TKI-naïve adults with locally advanced or metastatic non-small cell lung cancer (NSCLC) with activating EGFR mutation(s)	At RR	PCRS	3	N/A	No
9	Bosutinib	Adults with chronic phase, accelerated phase, and blast phase Ph + CML previously treated with one or more TKI(s) and for whom imatinib, nilotinib and dasatinib are not considered appropriate treatment options	Undocumented	PCRS	3	N/A	No
10	Decitabine	Adults aged 65 years and above with newly diagnosed de novo or secondary AML, according to the WHO classification, who are not candidates for standard induction chemotherapy	After RR	ODMS	1	N/A	No
11	Eribulin	LABC or MBC which has progressed after at least two chemotherapeutic regimens for advanced disease. Prior therapy should have included an anthracycline and a taxane unless patients were not suitable for these treatments	After HTA	ODMS	34	5	Yes
12	Pertuzumab	Adults with HER2-positive MBC or locally recurrent unresectable breast cancer, who have not received previous anti-HER2 therapy or chemotherapy for their metastatic disease	After HTA	ODMS	12	5	No
13	Ruxolitinib	Disease-related splenomegaly or symptoms in adults with post polycythaemia vera myelofibrosis	After HTA	PCRS	18	5	Yes
		Disease-related splenomegaly or symptoms in adults with primary myelofibrosis (chronic idiopathic myelofibrosis)					
		Disease-related splenomegaly or symptoms in adults with post essential thrombocythaemia myelofibrosis					
14	Aflibercept	Combination with irinotecan/5-fluorouracil/folinic acid (FOLFIRI) chemotherapy in adults with metastatic colorectal cancer that is resistant to or has progressed after an oxoliplatin-containing regimen	At HTA	Individual hospital budgets	12	5	No
15	Crizotinib	Adults with previously treated anaplastic lymphoma kinase (ALK)-positive advanced non-small cell lung cancer (NSCLC)	After HTA	PCRS	16	4	Yes
16	Vandetanib	Aggressive and symptomatic medullary thyroid cancer (MTC) in patients with unresectable locally advanced or metastatic disease	Undocumented	PCRS	11	N/A	No
17	Brentuximab vedotin	Adults with relapsed or refractory CD30+ Hodgkin lymphoma (HL): following autologous stem cell transplant (ASCT)	After HTA	ODMS	19	6	Yes
18	Brentuximab vedotin	Adults with relapsed or refractory CD30+ Hodgkin lymphoma (HL): following at least two prior therapies when ASCT or multi-agent chemotherapy is not an option	Undocumented	ODMS	19	NA	Yes
19	Brentuximab vedotin	Adults with relapsed or refractory systemic anaplastic large cell lymphoma (sALCL)	Undocumented	ODMS	19	NA	Yes
20	Enzalutamide	Adults with metastatic castration-resistant prostate cancer whose disease has progressed on or after docetaxel	After HTA	PCRS	13	7	Yes
21	Dabrafenib	Adults with unresectable or metastatic melanoma with the BRAF V600 mutation	At HTA	PCRS	11	7	No
22	Regorafenib	Adults unresectable or metastatic gastrointestinal stromal tumours (GIST) who progressed on or are intolerant to prior treatment with imatinib and sunitinib	Undocumented	PCRS	6	N/A	No
23	Regorafenib	Adults with metastatic colorectal cancer (mCRC) who have been previously treated with, or are not considered candidates for, available therapies. These include fluoropyrimidine-based chemotherapy, anti-VEGF and anti-EGFR therapies	After HTA	PCRS	18	4	No
24	Abiraterone	Metastatic castration resistant prostate cancer in men who are asymptomatic or mildly symptomatic after failure of androgen deprivation therapy in whom chemotherapy is not yet clinically indicated	After HTA	PCRS	27	5	Yes
25	Radium 223	Adults with castration-resistant prostate cancer, symptomatic bone metastases and no known visceral metastases	After HTA	ODMS	16	7	Yes
26	Obinutuzumab	Combination with chlorambucil for adults with previously untreated chronic lymphocytic leukaemia (CLL) and with comorbidities making them unsuitable for full-dose fludarabine based therapy	After HTA	ODMS	11	7	Yes
27	Pixantrone	Monotherapy for adults with multiply relapsed or refractory aggressive Non-Hodgkin B-cell Lymphomas (NHL)	At RR	ODMS	4	N/A	No
28	Siltuximab	Adults with Multicentric Castleman’s disease (MCD) who are human immunodeficiency virus (HIV) negative and human herpes virus 8 (HHV-8) negative	Undocumented	ODMS		N/A	NA
29	Trastuzumab Emtansine	Adults with HER2-positive, unresectable locally advanced or metastatic breast cancer who previously received trastuzumab and a taxane, separately or in combination. Patients should have either: received prior therapy for locally advanced or metastatic disease, or developed disease recurrence during or within 6 months of completing adjuvant therapy	After HTA	ODMS	21	5	Yes
30	Enzalutamide	Metastatic castrate resistant prostate cancer in men who are asymptomatic or mildly symptomatic after failure of androgen deprivation therapy (ADT) in whom chemotherapy is not yet clinically indicated	After HTA	PCRS	12	7	Yes
31	Lenvatinib	Adults with progressive, locally advanced or metastatic, differentiated (papillary/follicular/Hürthle cell) thyroid carcinoma (DTC), refractory to radioactive iodine	Undocumented	PCRS	6	N/A	No
32	Nab-Paclitaxel	Combination with gemcitabine for the first-line treatment of adults with metastatic adenocarcinoma of the pancreas	After HTA	ODMS	23	5	Yes
33	Pomalidomide	Combination with dexamethasone for adults with relapsed and refractory multiple myeloma who have received at least two prior treatment regimens, including both lenalidomide and bortezomib, and have demonstrated disease progression on the last therapy	After HTA	PCRS	28	6	Yes
34	Pembrolizumab 200/400 mg	First line monotherapy for advanced (unresectable or metastatic) melanoma in adults	At HTA	ODMS	10	6	No
35	Pembrolizumab 200/400 mg	Ipilimumab‐refractory patients with unresectable or advanced metastatic melanoma	After HTA	ODMS	10	5	Yes
36	Ibrutinib	Adults with relapsed or refractory mantle cell lymphoma	After HTA	PCRS	19	6	Yes
37	Ibrutinib	Adults with Waldenström’s macroglobulinaemia who have received at least one prior therapy, or in first line treatment for patients unsuitable for chemo‐immunotherapy	Undocumented	PCRS	N/A	N/A	NA
38	Ibrutinib	Adults with chronic lymphocytic leukaemia who have received at least one prior therapy, or in first line in the presence of 17p deletion or TP53 mutation in patients unsuitable for chemo‐immunotherapy	After HTA	PCRS	19	6	Yes
39	Ceritinib	Adults with anaplastic lymphoma kinase (ALK)-positive advanced non-small cell lung cancer (NSCLC) previously treated with crizotinib	After RR	PCRS	16	N/A	Yes
40	Ponatinib	Adults with Philadelphia chromosome positive acute lymphoblastic leukaemia (Ph + ALL) who are resistant to dasatinib; who are intolerant to dasatinib and for whom subsequent treatment with imatinib is not clinically appropriate; or who have the T315I mutation	After HTA	PCRS	36	7	No
41	Ponatinib	Adults with chronic phase chronic myeloid leukaemia (CML) who are resistant to dasatinib or nilotinib; who are intolerant to dasatinib or nilotinib and for whom subsequent treatment with imatinib is not clinically appropriate; or who have the T315I mutation	After HTA	PCRS	36	7	No
42	Ponatinib	Adults with accelerated phase chronic myeloid leukaemia (CML) who are resistant to dasatinib or nilotinib; who are intolerant to dasatinib or nilotinib and for whom subsequent treatment with imatinib is not clinically appropriate; or who have the T315I mutation	After HTA	PCRS	36	7	No
43	Ponatinib	Adults with blast phase chronic myeloid leukaemia (CML) who are resistant to dasatinib or nilotinib; who are intolerant to dasatinib or nilotinib and for whom subsequent treatment with imatinib is not clinically appropriate; or who have the T315I mutation	After HTA	PCRS	36	7	No
44	Idelalisib	Monotherapy for adults with follicular lymphoma (FL) that is refractory to two prior lines of treatment	Undocumented	PCRS	N/A	N/A	NA
45	Idelalisib	Combination with riTUXimab for adults with chronic lymphocytic leukaemia (CLL) who have received at least one prior therapy	After HTA	PCRS	24	8	Yes
46	Idelalisib	Combination with riTUXimab for adults with chronic lymphocytic leukaemia (CLL) as first line treatment in the presence of 17p deletion or TP53 mutation in patients who are not eligible for any other therapies	After HTA	PCRS	24	8	Yes
47	Idelalisib	Combination with Ofatumumab for adults with chronic lymphocytic leukaemia (CLL) who have received at least one prior therapy	Undocumented	PCRS	N/A	N/A	NA
48	Idelalisib	Combination with Ofatumumab for adults with chronic lymphocytic leukaemia (CLL) as first line treatment in the presence of 17p deletion or TP53 mutation in patients who are not eligible for any other therapies	Undocumented	PCRS	N/A	N/A	NA
49	Nintedanib	Combination with docetaxel for adults with locally advanced, metastatic of stage IIIB or IV, or locally recurrent NSCLC of adenocarcinoma tumour histology after first-line chemotherapy	After HTA	PCRS	24	5	Yes
50	Trifluridine and Tipracil	Adults with metastatic colorectal cancer (CRC) who have been previously treated with, or are not considered candidates for, available therapies including fluoropyrimidine-, oxaliplatin- and irinotecan-based chemotherapies, anti-VEGF agents, and anti-EGFR agents	After RR	PCRS	7	N/A	No
51	Nivolumab 240 mg	Monotherapy for adults with relapsed or refractory classical Hodgkin lymphoma (cHL) after autologous stem cell transplant (ASCT) and treatment with brentuximab vedotin	After RR	ODMS	8	N/A	Yes
52	Nivolumab 240/480 mg	As monotherapy for advanced (unresectable or metastatic) melanoma in adults (BRAF positive)	After HTA	ODMS	27	6	Yes
53	Nivolumab 240/480 mg	Monotherapy for advanced (unresectable or metastatic) melanoma in adults (BRAF negative)	After HTA	ODMS	27	6	Yes
54	Nivolumab 240/480 mg	Monotherapy for advanced renal cell carcinoma (RCC) after prior therapy in adults	After HTA	ODMS	17	7	Yes
55	Nivolumab Ipilimumab	Combination with ipilimumab for advanced (unresectable or metastatic) melanoma in adults	After HTA	ODMS	11	8	Yes
56	Alectinib	Adults with anaplastic lymphoma kinase (ALK)-positive advanced non-small cell lung cancer (NSCLC) previously treated with crizotinib	After HTA	PCRS	8	7	Yes
57	Obinutuzumab	Combination with bendamustine for patients with follicular lymphoma (FL) who did not respond or who progressed during or up to 6 months after treatment with rituximab or a rituximab-containing regimen	After HTA	ODMS	17	5	Yes
		Maintenance therapy in patients with follicular lymphoma (FL) who have responded to induction treatment with obinutuzumab and bendamustine or have stable disease					
58	Olaparib	Maintenance treatment of adults with platinum-sensitive relapsed BRCA-mutated (germline and/or somatic)—fallopian tube cancer who are in response (complete response or partial response) to platinum-based chemotherapy	After HTA	PCRS	32	5	Yes
		Maintenance treatment of adults with platinum-sensitive relapsed BRCA-mutated (germline and/or somatic)—high-grade serous epithelial ovarian cancer who are in response (complete response or partial response) to platinum-based chemotherapy					
		Maintenance treatment of adults with platinum-sensitive relapsed BRCA-mutated (germline and/or somatic)—primary peritoneal cancer who are in response (complete response or partial response) to platinum-based chemotherapy					
59	Vismodegib	Adults with local advanced basal cell carcinoma inappropriate for surgery or radiotherapy	After HTA	PCRS	53	5	Yes
60	Vismodegib	Adults with symptomatic metastatic basal cell carcinoma (MBCC)	After HTA	PCRS	53	5	Yes
61	Cobimetinib	Combination with vemurafenib for adults with unresectable or metastatic melanoma with a BRAF V600 mutation	After HTA	PCRS	23	6	Yes
62	Daratumumab	Monotherapy for adults with relapsed and refractory multiple myeloma whose prior therapy included a proteasome inhibitor and an immunomodulatory agent and who have demonstrated disease progression on the last therapy	After HTA	ODMS	22	6	Yes
63	Pembrolizumab 200 mg/400 mg	First-line for metastatic non-small cell lung carcinoma (NSCLC) in adults whose tumours express PD-L1 with a ≥ 50% tumour proportion score (TPS) with no EGFR or ALK positive tumour mutations	After HTA	ODMS	15	6	Yes
64	Trametinib	Combination with dabrafenib for adults with unresectable or metastatic melanoma with a BRAF V600 mutation	After HTA	PCRS	27	6	Yes
65	Nivolumab	Monotherapy for squamous cell cancer of the head and neck in adults progressing on or after platinum-based therapy	After RR	ODMS	11	N/A	Yes
66	Palbociclib	Hormone receptor (HR)-positive, human epidermal growth factor receptor 2 (HER2)-negative locally advanced or metastatic breast cancer in combination with fulvestrant in women who have received prior endocrine therapy (2nd line)	After HTA	PCRS	20	6	Yes
67	Palbociclib	Hormone receptor (HR)-positive, human epidermal growth factor receptor 2 (HER2)-negative locally advanced or metastatic breast cancer in combination with an aromatase inhibitor (1st line)	After HTA	PCRS	20	6	Yes
68	Carfilzomib	Carlfilzomib, lenalidomide and dexamethasone for adults with multiple myeloma who have received at least one pror therapy	After HTA	ODMS	32	6	Yes
69	Nivolumab	Monotherapy for locally advanced or metastatic non-small cell lung cancer (NSCLC) after prior chemotherapy in adults	After HTA	ODMS	38	6	Yes
70	Pembrolizumab 200 mg/400 mg	Monotherapy for adults with relapsed or refractory classical Hodgkin lymphoma (cHL) who are transplant-ineligible and have failed brentuximab vedotin	After RR	ODMS	10	N/A	Yes
71	Ixazomib	Combination with lenalidomide and dexamethasone for adults with multiple myeloma who have received at least one prior therapy	After HTA	PCRS	23	8	Yes
72	Venetoclax	Chronic lymphocytic leukaemia in the absence of 17p deletion or TP53 mutation in adults who have failed both chemoimmunotherapy and a B-cell receptor pathway inhibitor	After RR	PCRS	22	N/A	Yes
73	Venetoclax	Chronic lymphocytic leukaemia in the presence of 17p deletion or TP53 mutation in adults who are unsuitable for or have failed a B-cell receptor pathway inhibitor	After RR	PCRS	22	N/A	Yes
74	Cabozantinib	Advanced renal cell carcinoma in adults following prior VEGF targeted therapy	After HTA	PCRS	20	8	Yes
75	Ribociclib	Postmenopausal women with hormone receptor (HR) positive, human epidermal growth factor receptor 2 (HER2) negative locally advanced or metastatic breast cancer as initial endocrine-based therapy in combination with an aromatase inhibitor	after HTA	PCRS	16	6	Yes
76	Atezolizumab	Adults with locally advanced or metastatic non-small cell lung cancer after prior chemotherapy	After HTA	ODMS	17	5	Yes
77	Avelumab	Adults with metastatic Merkel cell carcinoma who have received 1 or more lines of chemotherapy for metastatic disease (1st line)	After HTA	ODMS	17	7	Yes
78	Avelumab	Adult with metastatic Merkel cell carcinoma who have received 1 or more lines of chemotherapy for metastatic disease (2nd line)	After HTA	ODMS	17	7	Yes
79	Blinatumomab	Adults with relapsed or refractory B cell precursor (BCP) Philadelphia chromosome negative acute lymphoblastic leukaemia (ALL) who have received no prior salvage treatment for relapsed/refractory disease and are considered eligible for transplant	After HTA	ODMS	40	5	Yes
80	Blinatumomab	Paediatric patients aged 1 year and older with Philadelphia chromosome negative B-cell precursor ALL which is refractory or in relapse after receiving at least two prior therapies or in relapse after receiving prior allogeneic hematopoietic stem cell transplantation	After RR	ODMS	3	N/A	Yes
81	Encorafenib and Binimetinib	Adults with advanced (unresectable or metastatic) melanoma with a BRAF V600 mutation	After RR	PCRS	3	N/A	Yes
82	Inotuzumab	Monotherapy for adults with relapsed or refractory CD22-positive B cell precursor acute lymphoblastic leukaemia (ALL). Adults with Philadelphia chromosome positive (Ph+) relapsed or refractory B cell precursor ALL should have failed treatment with at least 1 tyrosine kinase inhibitor (TKI)	After HTA	ODMS	20	6	Yes
83	Obinutuzumab	Combination with chemotherapy, followed by maintenance treatment in patients achieving a response, for previously untreated advanced follicular lymphoma (FL)	After HTA	ODMS	20	6	Yes
84	Alectinib	As monotherapy is indicated for the first-line treatment of adults with anaplastic lymphoma kinase (ALK)-positive advanced non-small cell lung cancer (NSCLC)	After HTA	PCRS	18	7	Yes
85	Brigatinib	Adults with anaplastic lymphoma kinase (ALK) positive advanced non-small cell lung cancer (NSCLC) previously treated with crizotinib	After RR	PCRS	6	N/A	Yes
86	Lorlatinib	Monotherapy for adults with anaplastic lymphoma kinase (ALK)-positive advanced non-small cell lung cancer (NSCLC), following disease progression on (i) alectinib or ceritinib as the first ALK-targeted treatment or (ii) crizotinib and at least one other ALK-targeted treatment	After RR	PCRS	3	N/A	Yes
87	Tivozanib	First line for adults with advanced renal cell carcinoma (RCC) and for adults who are VEGFR and mTOR pathway inhibitor-naïve following disease progression after one prior treatment with cytokine therapy for advanced RCC	After RR	PCRS	8	N/A	Yes
88	Dacomitinib	Dacomitinib (Vizimpro®) as monotherapy, for the first-line treatment of adults with locally advanced or metastatic non-small cell lung cancer (NSCLC) with epidermal growth factor receptor (EGFR)-activating mutations	After RR	PCRS	6	N/A	Yes
89	Osimertinib	Adults with locally advanced or metastatic epidermal growth factor receptor (EGFR) T790M mutation-positive non-small cell lung cancer (NSCLC)	After HTA	PCRS	23	5	Yes
90	Pertuzumab	Pertuzumab in combination with trastuzumab and chemotherapy for the neoadjuvant treatment of adults with HER2-positive, locally advanced, inflammatory, or early stage breast cancer at high risk of recurrence	After HTA	ODMS	25	8	No
91	Venetoclax	Combination with rituximab for adults with chronic lymphocytic leukaemia (CLL) who have received at least one prior therapy	After HTA	PCRS	21	8	Yes

**Table 7:** Available cost-effectiveness evidence

Pair number	Drug name	Indication	Basecase ICER	Costs and effects reported for all strategies	Incremental costs, €	Incremental effects, QALYs	Gross 5-year budget impact, €M
1	Ipilimumab	Adults with advanced (unresectable or metastatic) malignant melanoma	147,899	None	NA	NA	6.75
2	Abiraterone	Metastatic castration resistant prostate cancer which has progressed on or after a docetaxel-based chemotherapy regimen	160,388	None	NA	NA	9.84
5	Cabazitaxel	Metastatic castration resistant prostate cancer previously treated with docetaxel containing regimen	120,084	None	NA	NA	5.60
7	Vemurafenib	Adults with BRAF V600 mutation-positive unresectable or metastatic melanoma	131,883	None	NA	NA	12.10
11	Eribulin	LABC or MBC which has progressed after at least two chemotherapeutic regimens for advanced disease. Prior therapy should have included an anthracycline and a taxane unless patients were not suitable for these treatments	76,610	None	NA	NA	5.40
12	Pertuzumab	Adults with HER2-positive MBC or locally recurrent unresectable breast cancer, who have not received previous anti-HER2 therapy or chemotherapy for their metastatic disease	206,720	None	NA	NA	39.37
13	Ruxolitinib	Disease-related splenomegaly or symptoms in adults with post polycythaemia vera myelofibrosis	70,252	All	84,292	1.20	NA
		Disease-related splenomegaly or symptoms in adults with primary myelofibrosis (chronic idiopathic myelofibrosis)					
		Disease-related splenomegaly or symptoms in adults with post essential thrombocythaemia myelofibrosis					
14	Aflibercept	Combination with irinotecan/5-fluorouracil/folinic acid (FOLFIRI) chemotherapy in adults with metastatic colorectal cancer that is resistant to or has progressed after an oxoliplatin-containing regimen	64,132	All	15,410	0.24	5.94
15	Crizotinib	Adults with previously treated anaplastic lymphoma kinase (ALK)-positive advanced non-small cell lung cancer (NSCLC)	165,616	Some	41,690	0.25	6.28
17	Brentuximab vedotin	Adults with relapsed or refractory CD30+ Hodgkin lymphoma (HL): following autologous stem cell transplant (ASCT)	78,106	Some	85,786	1.10	5.53
20	Enzalutamide	Adults with metastatic castration-resistant prostate cancer whose disease has progressed on or after docetaxel	98,949	None	NA	NA	NA
21	Dabrafenib	Adults with unresectable or metastatic melanoma with the BRAF V600 mutation	84,473	Some	113,613	1.35	7.10
23	Regorafenib	Adults with metastatic colorectal cancer (mCRC) who have been previously treated with, or are not considered candidates for, available therapies. These include fluoropyrimidine-based chemotherapy, anti-VEGF and anti-EGFR therapies	126,246	All	12,653	0.10	4.00
24	Abiraterone	Metastatic castration resistant prostate cancer in men who are asymptomatic or mildly symptomatic after failure of androgen deprivation therapy in whom chemotherapy is not yet clinically indicated	171,384	Some	85,466	0.50	NA
25	Radium 223	Adults with castration-resistant prostate cancer, symptomatic bone metastases and no known visceral metastases	80,361	None	NA	NA	5.90
26	Obinutuzumab	Combination with chlorambucil for adults with previously untreated chronic lymphocytic leukaemia (CLL) and with comorbidities making them unsuitable for full-dose fludarabine based therapy	67,409	Some	15,504	0.23	7.60
29	Trastuzumab Emtansine	Adults with HER2-positive, unresectable locally advanced or metastatic breast cancer who previously received trastuzumab and a taxane, separately or in combination. Patients should have either: received prior therapy for locally advanced or metastatic disease, or developed disease recurrence during or within 6 months of completing adjuvant therapy	98,809	None	NA	NA	19.74
30	Enzalutamide	Metastatic castrate resistant prostate cancer in men who are asymptomatic or mildly symptomatic after failure of androgen deprivation therapy (ADT) in whom chemotherapy is not yet clinically indicated	126,709	All	84,634	0.67	71.38
32	Nab-Paclitaxel	Combination with gemcitabine for the first-line treatment of adults with metastatic adenocarcinoma of the pancreas	73,867	Some	10,553	0.15	4.50
33	Pomalidomide	Combination with dexamethasone for adults with relapsed and refractory multiple myeloma who have received at least two prior treatment regimens, including both lenalidomide and bortezomib, and have demonstrated disease progression on the last therapy	102,485	Some	59,527	0.58	15.20
34	Pembrolizumab 200/400 mg	First line monotherapy for advanced (unresectable or metastatic) melanoma in adults	Dominant	None	-3,092	0.42	63.00
35	Pembrolizumab 200/400 mg	Ipilimumab‐refractory patients with unresectable or advanced metastatic melanoma	85,766	Some	72,280	0.84	1.80
36	Ibrutinib	Adults with relapsed or refractory mantle cell lymphoma	89,931	Some	33,010	0.37	7.00
38	Ibrutinib	Adults with chronic lymphocytic leukaemia who have received at least one prior therapy, or in first line in the presence of 17p deletion or TP53 mutation in patients unsuitable for chemo‐immunotherapy	82,786	Some	243,725	2.94	32.00
40	Ponatinib	Adults with Philadelphia chromosome positive acute lymphoblastic leukaemia (Ph + ALL) who are resistant to dasatinib; who are intolerant to dasatinib and for whom subsequent treatment with imatinib is not clinically appropriate; or who have the T315I mutation	42,000	None	NA	NA	NA
41	Ponatinib	Adults with chronic phase chronic myeloid leukaemia (CML) who are resistant to dasatinib or nilotinib; who are intolerant to dasatinib or nilotinib and for whom subsequent treatment with imatinib is not clinically appropriate; or who have the T315I mutation	20,000	None	NA	NA	NA
42	Ponatinib	Adults with accelerated phase chronic myeloid leukaemia (CML) who are resistant to dasatinib or nilotinib; who are intolerant to dasatinib or nilotinib and for whom subsequent treatment with imatinib is not clinically appropriate; or who have the T315I mutation	Dominant	None	NA	NA	NA
43	Ponatinib	Adults with blast phase chronic myeloid leukaemia (CML) who are resistant to dasatinib or nilotinib; who are intolerant to dasatinib or nilotinib and for whom subsequent treatment with imatinib is not clinically appropriate; or who have the T315I mutation	20,000	None	NA	NA	NA
45	Idelalisib	Combination with riTUXimab for adults with chronic lymphocytic leukaemia (CLL) who have received at least one prior therapy	57,440	All	102,325	1.78	27.51
46	Idelalisib	Combination with riTUXimab for adults with chronic lymphocytic leukaemia (CLL) as first line treatment in the presence of 17p deletion or TP53 mutation in patients who are not eligible for any other therapies	71,388	All	NA	NA	NA
49	Nintedanib	Combination with docetaxel for adults with locally advanced, metastatic of stage IIIB or IV, or locally recurrent NSCLC of adenocarcinoma tumour histology after first-line chemotherapy	72,751	None	NA	NA	0.22
52	Nivolumab 240/480 mg	As monotherapy for advanced (unresectable or metastatic) melanoma in adults (BRAF positive)	101,282	None	NA	NA	98.80
53	Nivolumab 240/480 mg	Monotherapy for advanced (unresectable or metastatic) melanoma in adults (BRAF negative)	76,540	None	NA	NA	
54	Nivolumab 240/480 mg	Monotherapy for advanced renal cell carcinoma (RCC) after prior therapy in adults	55,864	All	63,110	1.44	26.73
55	Nivolumab Ipilimumab	Combination with ipilimumab for advanced (unresectable or metastatic) melanoma in adults	47,748	Some	101,354	2.12	61.00
56	Alectinib	Adults with anaplastic lymphoma kinase (ALK)-positive advanced non-small cell lung cancer (NSCLC) previously treated with crizotinib	178,358	Some	99,169	0.56	13.60
57	Obinutuzumab	Combination with bendamustine for patients with follicular lymphoma (FL) who did not respond or who progressed during or up to 6 months after treatment with rituximab or a rituximab-containing regimen	52,248	Some	60,142	1.15	6.15
		Maintenance therapy in patients with follicular lymphoma (FL) who have responded to induction treatment with obinutuzumab and bendamustine or have stable disease					
58	Olaparib	Maintenance treatment of adults with platinum-sensitive relapsed BRCA-mutated (germline and/or somatic)—fallopian tube cancer who are in response (complete response or partial response) to platinum-based chemotherapy	111,248	All	93,447	0.84	4.86
		Maintenance treatment of adults with platinum-sensitive relapsed BRCA-mutated (germline and/or somatic)—high-grade serous epithelial ovarian cancer who are in response (complete response or partial response) to platinum-based chemotherapy					
		Maintenance treatment of adults with platinum-sensitive relapsed BRCA-mutated (germline and/or somatic)—primary peritoneal cancer who are in response (complete response or partial response) to platinum-based chemotherapy					
59	Vismodegib	Adults with local advanced basal cell carcinoma inappropriate for surgery or radiotherapy	556,657	None	NA	NA	NA
60	Vismodegib	Adults with symptomatic metastatic basal cell carcinoma (MBCC)	240,902	None	NA	NA	NA
61	Cobimetinib	Combination with vemurafenib for adults with unresectable or metastatic melanoma with a BRAF V600 mutation	326,868	Some	168,266	0.51	22.10
62	Daratumumab	Monotherapy for adults with relapsed and refractory multiple myeloma whose prior therapy included a proteasome inhibitor and an immunomodulatory agent and who have demonstrated disease progression on the last therapy	127,785	None	39,334	0.31	17.60
63	Pembrolizumab 200 mg/400 mg	First-line for metastatic non-small cell lung carcinoma (NSCLC) in adults whose tumours express PD-L1 with a ≥ 50% tumour proportion score (TPS) with no EGFR or ALK positive tumour mutations	96,376	Some	105,811	1.10	65.30
64	Trametinib	Combination with dabrafenib for adults with unresectable or metastatic melanoma with a BRAF V600 mutation	244,822	Some	182,417	0.75	24.80
66	Palbociclib	Hormone receptor (HR)-positive, human epidermal growth factor receptor 2 (HER2)-negative locally advanced or metastatic breast cancer in combination with fulvestrant in women who have received prior endocrine therapy (2nd line)	256,993	Some	63,306	0.25	78.64
67	Palbociclib	Hormone receptor (HR)-positive, human epidermal growth factor receptor 2 (HER2)-negative locally advanced or metastatic breast cancer in combination with an aromatase inhibitor (1st line)	217,312	Some	116,925	0.54	
68	Carfilzomib	Carlfilzomib, lenalidomide and dexamethasone for adults with multiple myeloma who have received at least one pror therapy	125,759	Some	107,801	0.86	26.40
69	Nivolumab	Monotherapy for locally advanced or metastatic non-small cell lung cancer (NSCLC) after prior chemotherapy in adults	202,393	Some	88,117	0.44	57.10
71	Ixazomib	Combination with lenalidomide and dexamethasone for adults with multiple myeloma who have received at least one prior therapy	703,426	Some	NA	NA	39.30
74	Cabozantinib	Advanced renal cell carcinoma in adults following prior VEGF targeted therapy	208,156	Some	68,960	0.33	16.30
75	Ribociclib	Postmenopausal women with hormone receptor (HR) positive, human epidermal growth factor receptor 2 (HER2) negative locally advanced or metastatic breast cancer as initial endocrine-based therapy in combination with an aromatase inhibitor	220,591	All	41,816	0.14	16.02
76	Atezolizumab	Adults with locally advanced or metastatic non-small cell lung cancer after prior chemotherapy	152,458	Some	60,710	0.40	38.73
77	Avelumab	Adults with metastatic Merkel cell carcinoma who have received 1 or more lines of chemotherapy for metastatic disease (1st line)	130,984	Some	82,319	1.40	2.10
78	Avelumab	Adult with metastatic Merkel cell carcinoma who have received 1 or more lines of chemotherapy for metastatic disease (2nd line)	54,540	Some	77,213	1.84	
79	Blinatumomab	Adults with relapsed or refractory B cell precursor (BCP) Philadelphia chromosome negative acute lymphoblastic leukaemia (ALL) who have received no prior salvage treatment for relapsed/refractory disease and are considered eligible for transplant	472,215	Some	104,693	0.22	9.20
82	Inotuzumab	Monotherapy for adults with relapsed or refractory CD22-positive B cell precursor acute lymphoblastic leukaemia (ALL). Adults with Philadelphia chromosome positive (Ph +) relapsed or refractory B cell precursor ALL should have failed treatment with at least 1 tyrosine kinase inhibitor (TKI)	68,568	Some	84,064	1.23	5.82
83	Obinutuzumab	Combination with chemotherapy, followed by maintenance treatment in patients achieving a response, for previously untreated advanced follicular lymphoma (FL)	95,606	Some	43,809	0.46	29.10
84	Alectinib	As monotherapy is indicated for the first-line treatment of adults with anaplastic lymphoma kinase (ALK)-positive advanced non-small cell lung cancer (NSCLC)	146,721	Some	98,979	0.67	13.60
89	Osimertinib	Adults with locally advanced or metastatic epidermal growth factor receptor (EGFR) T790M mutation-positive non-small cell lung cancer (NSCLC)	115,912	All	78,556	0.68	50.23
90	Pertuzumab	Pertuzumab in combination with trastuzumab and chemotherapy for the neoadjuvant treatment of adults with HER2-positive, locally advanced, inflammatory, or early stage breast cancer at high risk of recurrence	75,400	All	40,734	0.54	52.36
91	Venetoclax	Combination with rituximab for adults with chronic lymphocytic leukaemia (CLL) who have received at least one prior therapy	96,130	Some	120,024	1.25	42.00

**Table 8:** Number of approvals and reported 5-year gross budget impact by market authorisation holder

Market authorisation holder	Approvals, *n*	5-year gross budget impact, €M
Roche	13	254.5
Bristol-Myers Squibb	8	250.4
Janssen-Cilag	8	66.4
Pfizer	7	90.7
Takeda	6	44.8
Gilead	5	27.5
Novartis	5	47.9
Bayer	4	15.8
Incyte	4	NA
Merck Sharp & Dohme	4	130.1
AbbVie	3	42.0
Amgen	3	35.6
Les Laboratoires Servier	3	NA
Astellas	2	71.4
AstraZeneca	2	55.1
Boehringer Ingelheim	2	0.2
Celgene	2	19.7
Eisai	2	5.4
Merck	2	2.1
EUSA Pharma	1	NA
Genzyme	1	NA
Ipsen	1	16.3
Nordic Group	1	NA
Pierre Fabre Médicament	1	NA
Sanofi-Aventis	1	5.6
Total	91	1181.6
